# Design, Synthesis, In Silico Testing, and In Vitro Evaluation of Thiazolidinone-Based Benzothiazole Derivatives as Inhibitors of α-Amylase and α-Glucosidase

**DOI:** 10.3390/ph15101164

**Published:** 2022-09-20

**Authors:** Shoaib Khan, Shahid Iqbal, Marwa Khan, Wajid Rehman, Mazloom Shah, Rafaqat Hussain, Liaqat Rasheed, Yousaf Khan, Ayed A. Dera, Rami Adel Pashameah, Eman Alzahrani, Abd-ElAziem Farouk

**Affiliations:** 1 Department of Chemistry, Hazara University, Mansehra 21120, Pakistan; 2 Department of Chemistry, School of Natural Sciences (SNS), National University of Science and Technology (NUST), H-12, Islamabad 46000, Pakistan; 3 Department of Chemistry, Abbottabad University of Science and Technology (AUST), Abbottabad 22500, Pakistan; 4 Department of Chemistry, COMSATS University Islamabad Campus, Islamabad 45550, Pakistan; 5 Department of Clinical Laboratory Sciences, College of Applied Medical Sciences, King Khalid University, Abha 61421, Saudi Arabia; 6 Department of Chemistry, Faculty of Applied Science, Umm Al-Qura University, Makkah 24230, Saudi Arabia; 7 Department of Chemistry, College of Science, Taif University, P.O. Box 11099, Taif 21944, Saudi Arabia; 8 Department of Biotechnology, College of Science, Taif University, P.O. Box 11099, Taif 21944, Saudi Arabia

**Keywords:** synthesis, α-amylase and α-glucosidase enzymes, thiazolidinone, benzothiazole, SAR, molecular docking

## Abstract

In this study, a stepwise reaction afforded thiazolidinone-based benzothiazole derivatives **1**–**15**, and the synthesized derivatives were then screened for biological significance and found to be the leading candidates against α-amylase and α-glucosidase enzymes. Almost all derivatives showed excellent to good activity ranging against α-amylase, IC50 = 2.10 ± 0.70 to 37.50 ± 0.70 μM, and α-glucosidase, IC50 = 3.20 ± 0.05 to 39.40 ± 0.80 μM. Some analogues such as **4** (2.40 ± 0.70 and 3.50 ± 0.70 μM), **5** (2.30 ± 0.05 and 4.80 ± 0.10 μM), and **6** (2.10 ± 0.70 and 3.20 ± 0.70 μM) were found with folds better activity than that of the standard drug acarbose (9.10 ± 0.10 and 10.70 ± 0.10 μM), respectively. Moreover, the structure–activity relationship (SAR) has been established for all compounds. A molecular docking study has been carried out to explore the binding interactions against α-amylase and α-glucosidase enzymes.

## 1. Introduction

Insulin deficiency due to altered carbohydrate and lipid metabolism rates is associated with mortality and the highest healthcare costs. It is a non-communicable disease that affects approximately 387 million people, while 4.9 million people died from diabetes in 2014 [1]. Diabetic patients have the highest blood glucose level after taking food; this condition is also called postprandial hyperglycemia (PPHG). Numerous serious complications are caused by diabetes mellitus such as cardiovascular disease, neuropathy, retinopathy, etc. [2]. The main cause of this disease is the detrimental action of two enzymes, α-amylase and α-glucosidase, which break down glycogen into glucose in a series of reactions. First, α-amylase breaks down larger carbohydrates, including glycogen, into starch and maltose, which are further broken down into simpler glucose units by α-glucosidase, raising blood sugar levels [3]. Many drugs on the market are used to block the function of these enzymes, allowing some control over blood sugar levels [4].

Among the few drugs are voglibose, miglitol, and acarbose. These drugs are used by most people around the world to prevent the breaking of macromolecules into simple sugar units. Acarbose is one of the major drugs used against both α-amylase and α-glucosidase infections, while the remaining drugs, such as voglibose and miglitol, are only effective against α-glucosidase. However, all these drugs have some serious gastrointestinal side effects [5,6]. These drugs are very expensive, while most people in the world live below the poverty line [7]. Therefore, researchers in different parts of the world try to attempt an inhibitor that can be proven as a potent inhibitor against both α-amylase and α-glucosidase enzymes by using different sources such as marine algae, bacteria, fungi, and plants [8,9,10,11]. A few of them studied the crude extracts (organic or aqueous), while few of them also studied pure compounds [12,13]. Most of the pure compounds and extracts were found to be efficient against both these enzymes but had some complications [14,15].

**Figure 1:**
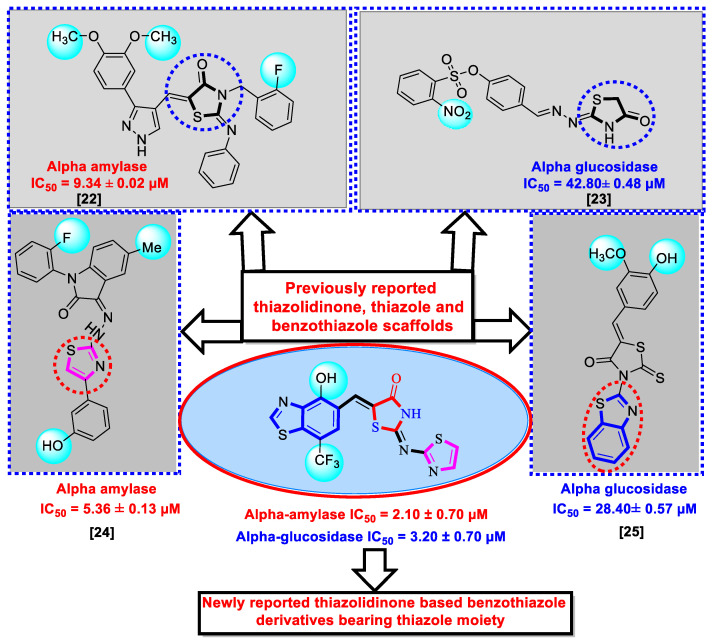
Rational of the current study, refs [22,23,24,25].

Heterocyclic derivatives have been proven as a potent inhibitor for different infections due to their efficient and excellent biological profile. Most of the heterocyclic class of compounds was found in more than one inhibitory potential. Being a core drug component, these heterocyclic classes of analogs have received substantial attention in the past few decades because of their diverse applications in the field of medicine. In this regard, the thiazolidinedione analogue also preferred as well-known heterocyclic derivative containing different hetero-atom being membered of the ring. The potent nature of the thiazolidinone derivative exhibits versatile behavior with different drug candidates and has a broad spectrum of biological applications including anti-bacterial [16], anti-fungal [17], anti-viral [18], anti-inflammatory [19], anti-tuberculosis [20], etc. Benzohydrazide-based imines and 4-thiazolidinone analogs [21] are used as α-amylase and α-glucosidase inhibitors. The present study also described the basic skeleton and function of thiazolidinone-based benzothiazole derivatives as an effective inhibitor against α-amylase and α-glucosidase enzymes, when compared with the standard drug acarbose. Moreover, rational of the current study also discussed for comparison of newly and pre-vious reported scaffolds as shown in Figure 1.

## 2. Results and Discussion

### 2.1. Chemistry

**Scheme 1:**
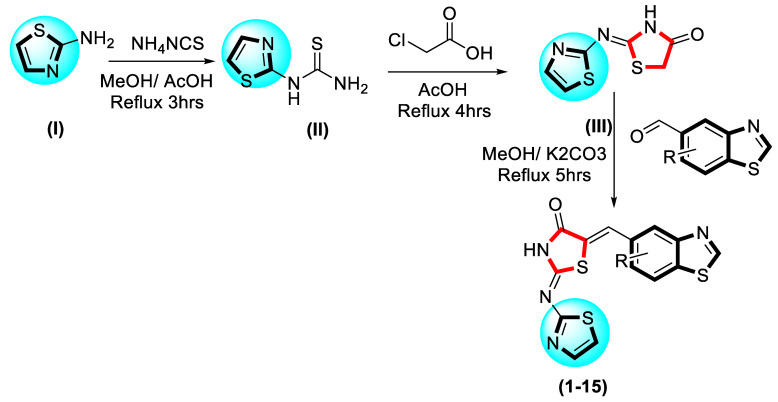
Synthesis of thiazolidinedione-based benzothiazole derivatives **1**–**15**.

A series of chemical reactions were conducted stepwise for the synthesis of thiazolidinone-based benzothiazole derivatives by using 2-aminothiazole **I**; ammonium isothiocyanate was dissolved in methanol and refluxed in the reaction mixture in the presence of glacial acetic acid to yields thiazole-based thiourea analogue **II**, which was then treated with chloroacetic acid under reflux in acidic medium, and afforded thiazole-based thiazolidinone derivatives **III**. In the next step, compounds **III** were further subjected along with different substituted benzothiazole-bearing aldehyde derivatives in methanol, and the reaction mixture was refluxed in the presence of potassium carbonate to yield thiazolidinone-based chalcone derivatives **1**–**15** as shown in Scheme 1. 

#### Spectroscopic Characterization and Elucidation of Derivative-**1**

**Figure 2:**
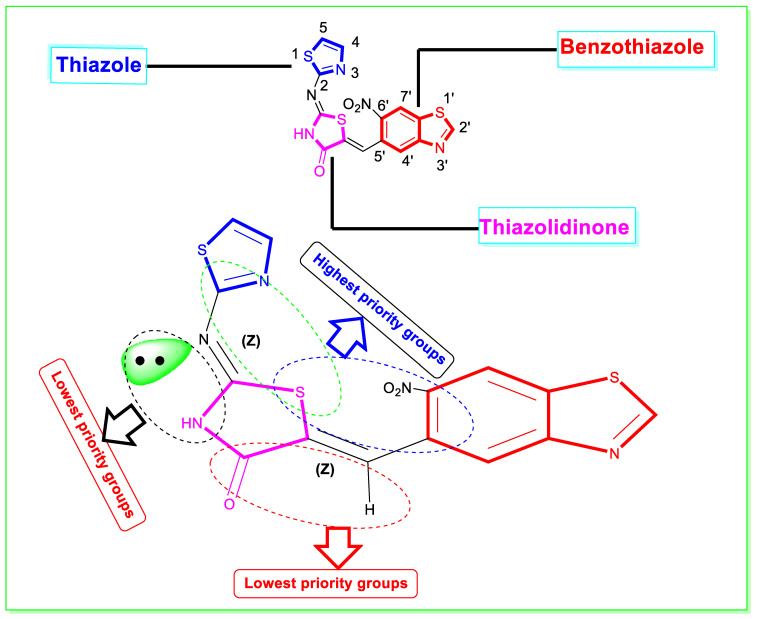
The general representation of (2Z,5Z)-5-((5-nitrobenzo [d]thiazol-6-yl) methylene)-2-(thiazol-2-ylimino)thiazolidin-4-one (**1**).

Among the following synthesized compounds, one of the derivatives has been mentioned in such a way to prove the correctness of the name and structure of the compound, which is given below in Figure 2. Among these heterocyclic compounds, benzothiazole possesses a different functional group, by which the structure and its spectra are different from one another. The synthesized derivative-1 is generally divided into three sections, and their ^1^H and ^13^C values are analyzed in the coupling range and through ^1^HNMR, where the singlet for the NH proton of thiazolidinone appears in the range of 11.65–11.69 ppm, with 9.37 showing a singlet for (C=N, 2′), 8.34 showing a singlet for CH (7′), 8.26 a singlet for CH (4′), 7.33 a singlet for CH (proton appears between benzothiazole and thiazolidinone), 7.13 showing a doublet for CH (4-thiazole proton), and 6.84 showing a doublet for CH (5-thiazole proton). The proton spectra clearly represent the structure having different hetero-atoms, through which the value of the proton appeared at different ranges. Likewise, the ^13^C value was also in varied ranges due to the presence of the hetero-atom in this regard at 162.2 ppm, which was shown by the carbonyl carbon of the thiazolidinone ring at 149.3 ppm, showing the thiazole carbon directly attached with two nitrogen atoms. Next, the carbon appeared at 148.8 ppm of the thiazolidinone ring, attached to a double bond nitrogen and emerged in both the nitrogen and sulfur of the thiazolidinone ring, with the 148.4 ppm range showing the carbon of the benzothiazole (2′), likewise at 127.4 ppm the carbon of the benzothiazole (fused carbon attached to a nitrogen atom) appeared. Then, carbon appeared at 125.0 ppm in between the thiazolidinone and benzothiazole rings, with 123.4 ppm carbon of the benzothiazole (5′) attached to –NO_2_. Carbon (4) appeared at 122.2 ppm attached to the nitrogen of the thiazole ring, while at 121.1 ppm the value is shown by the fused carbon of the benzothiazole near to the sulfur atom. Moreover, at 119.7 ppm the carbon of the benzothiazole (6′) appeared and at 118.8 ppm of the benzothiazole carbon (7′) appeared, at 115.6 ppm the carbon of the thiazole (5) appeared, at 109.0 ppm it was showing the carbon of the benzothiazole (4′), and at 100.5 ppm the carbon of the thiazolidinone appeared, which is linked through a double bond toward the benzothiazole. The spectral analysis of synthesized derivatives clearly identifies the existence of proton and carbon in a varied range with different coupling constants, as shown in Figure 2. In addition, NMR spectral study of the different compounds (in deuterated dimethyl sulfoxide DMSO-d_6_) showed that these compounds exist in the form of a (Z, Z) conformation, as the highest priority groups are present on same side of both double bonds. Compound structures were confirmed by spectroscopic analyses ^1^H, ^13^C NMR, and high-resolution mass spectrometry (HRMS). General representation of synthesized molecule as shown in Figure 2.

### 2.2. In Vitro α-Amylase and α-Glucosidase Inhibitory Activities

The entire synthesized compounds were screened for α-amylase and α-glucosidase inhibitory activities in order to investigate their potency and binding interaction with the active sites of enzymes (Table 1). Biological profile of all the candidates were obtained through experimental-based analysis, as described in the inhibitory assay (**5.3** and **5.4**). Varied substituted compounds were compared by means of the attachment of the same substituents on the phenyl ring and their potency with the standard drug acarbose.

**Figure 3:**
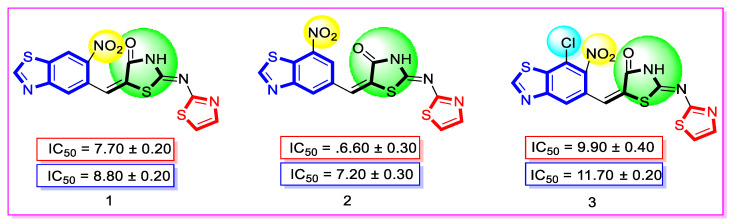
SAR study of analogues **1**, **2**, and **3**.

The phenyl ring contained different substituents such as a nitro group at the *ortho-* and *meta-*position. The phenyl ring attached to the thiazolidinone ring, as in the case of **1**, **2**, and **3**, has been identified and showed significant results against *α*-amylase and α-glucosidase, but derivative-**1** was found to be one and three folds better than **2** and **3** as well as **5** folds more active than the standard acarbose as reference inhibitor. Among nitro-substituted compounds, the derivative-**1** having a nitro group at the *ortho*-position exhibited excellent potential for both *α*-amylase and *α*-glucosidase. Comparison criteria were set for those analogs bearing a nitro group at *meta*-position **2**, and a little bit of decrease in *α*-amylase and α-glucosidase potential was observed, which might be the position of substituents at the *ortho*-position and *meta*-position. By this consideration, it was concluded that substitution of a nitro group at the *meta*-position of the phenyl ring decreases the interactive property of the molecule. The activity of analog-**3** decreased due to the presence of a chlorine group, which causes steric hindrance. In this stage, analogue 1 showed better potential against *α*-amylase and *α*-glucosidase as shown in Figure 3. 

**Figure 4:**
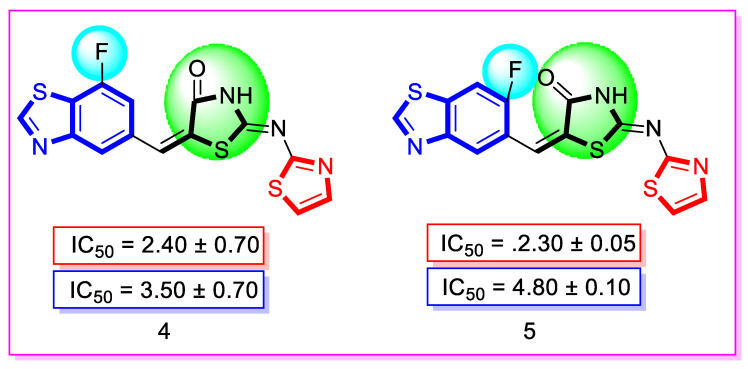
SAR study of analogues **4** and **5**.

Analogues **4** and **5** bore fluoro groups at the *meta-* and *ortho*-position, respectively, and exhibited the highest potential against *α*-amylase and *α*-glucosidase in comparison to acarbose. The effective nature of analogues **4** and **5** against *α*-amylase and *α*-glucosidase activity might be due to the presence of two flouro groups, which interact better with the active site of the enzyme through series of hydrogen bonds. Moreover, the position of the fluoro groups around the phenyl ring has a significant effect to increase the biological activity of analogues. Thus, analogue **5** bore a flouro group at the *ortho*-position of phenyl ring and showed excellent inhibitory potential than the *meta-*substituted analogue **4**, which was considered as the top-ranked analogue, or the series was found with a few folds better potential than acarbose as shown in Figure 4. 

**Figure 5:**
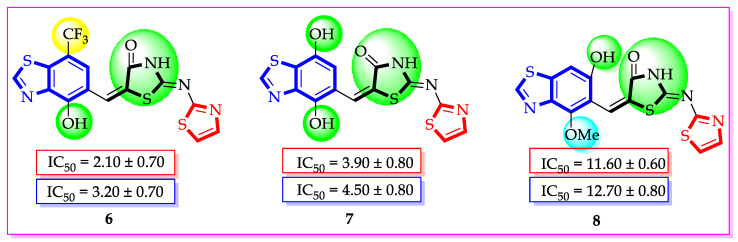
SAR study of analogues **6**, **7**, and **8**.

Analogues **6**, **7**, and **8** bore different numbers of hydroxyl group at *ortho* **6**, *ortho/meta* **7**, and *ortho-*position **8** and showed strong interaction against *α*-amylase and *α*-glucosidase as shown in Figure 5. Two analogues were found with a few folds better activity than acarbose. In this regard, analogue **7** was found with excellent potential when compared with the acarbose drug. Analogue **7** bore two –OH groups and showed a superior inhibitory profile due to strong hydrogen bonding; therefore, it is ranked first in this group, while analogue **6** bore one –-OH group and was found with fewer smaller interactions than analogue **7**, so it might be the presence of CF_3_**.** However, analogue **8** was found to have comparable activity with acarbose, so the lower activity might be due to the presence of the methoxy substituent, which increases steric hindrance and, thus, reduces the biological potential. 

**Figure 6:**
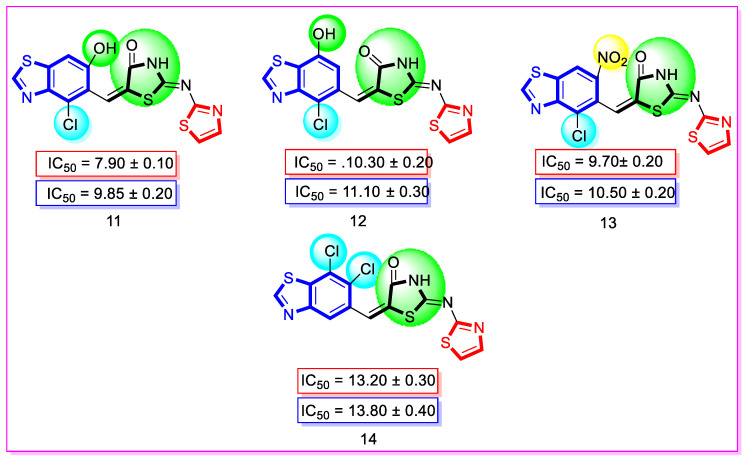
SAR study of analogues **11**, **12**, **13**, and **14**.

Analogues having either hydroxyl groups or hydroxyl groups along with -Cl group have emerged as potent inhibitors of both α-amylase and α-glucosidase enzymes. Analogue **11**, bearing *meta*-OH and *ortho*-Cl groups, was recognized as the most active analogue among the hydroxyl groups bearing the potency of analogue **11**, which was reduced by the introduction of one –OH group on the *meta*-position instead of *ortho-*position, as in analogue **12**, which might cause steric hindrance. This is because of the presence of a -Cl group at the *ortho*-site, which decreases the enzymatic activity. The activity of analogue **11** was further reduced due to the presence of one -Cl group, which might reduce the binding interactions, as in analogue **13**, and two -Cl, as in analogue **14**, which might also decrease the biological profile of the molecule as shown in Figure 6.

Some of the screened analogues were found with moderate to the least potential against both α-amylase and α-glucosidase enzymes. When compared with the standard drug acarbose (10.20 ± 0.10 and 11.70 ± 0.10), these analogues displayed a varied range of inhibitory profiles in both activities, such as analogues **9** (21.50 ± 0.30 and 23.20 ± 0.40), **10** (23.20 ± 0.10 and 24.60 ± 0.20), and **15** (27.50 ± 0.70 and 28.40 ± 0.80). The moderate and least potent behavior of the synthesized derivatives may be due to the presence of electron-withdrawing or bulky groups that reduce the possibility of interactions with protein-active sites. In the present study, bromine-substituted analogues were found to be less active, due to the bulky nature that inhibits ligand interactions.

Overall, the results showed that the inhibitory profile of analogues mostly depends on the attached group, which either enhances or decreases the biological potential of the ligands, and their nature can be identified. Most of the effect is shown by electron-donating and electron-withdrawing groups, which change the biological nature of molecules; therefore, a strong interaction profile is mainly obtained when an electron-donating group is present, which increases the charge in the aromatic ring, since it increases interactions, while in the case of an electron-withdrawing group, the activity profile was observed as somewhat less, due to the least number of ligand protein interactions. The above screened derivatives are also based on such phenomena, due to which the most potent molecules were obtained bearing a hydroxyl group, which increases the nucleophilic character and the chances of interactions with the active site of enzymes and also increases through hydrogen bonding. It was clearly observed in the molecular docking study, which revealed the binding modalities of the ligand molecule were uniformly spread in 2D and 3D structures.

### 2.3. Molecular Docking

The molecular docking study reveals the binding modalities of the ligand molecule with the active sites of enzymes, through different bonding interactions. Ligands strongly interact with the active sites of enzymes due to attached groups, and their pi-electronic system is also involved with the electron-deficient sites of enzymes. By this interaction, the inhibition criteria take place. Molecular docking studies express those amino acids that have binding sites where ligands interact and fit to their receptor sites.

In the activity section, the subjected molecule was then carried out for the molecular docking study, to explore the binding mode of interactions with the active sites of enzymes. For this purpose, varied software was utilized such as Discovery Studio Visualizer (DSV) and AutoDock Vina1.5.7 [26,27,28,29], and protein was retrieved from an online source such as the Protein Data Bank (PDB). The designated codes for α-amylase and α-glucosidase enzyme are 1b2y and 3w37, respectively.

A stepwise procedure was used to find out the basic interactions of molecules in the enzyme complex. The primary need of docking is to maintain and prepare protein and ligand molecules, by removing water molecules and adding polar hydrogen and required charges such as Kollman and Gasteiger. Both the protein and ligand molecule were saved in PDBQT format in the docking folder, and the XYZ coordinates were saved. The location of the folder is mentioned in the command prompt, which is an easy way to complete the docking procedure, through which the best pose was identified. In total, **9** poses were generated, in which the best one having a very small binding affinity was identified, and their 2D and 3D structure coordinate was designed in DSV. The protein–ligand interactions (PLI) are summarized below.

#### Docking Results

**Figure 7:**
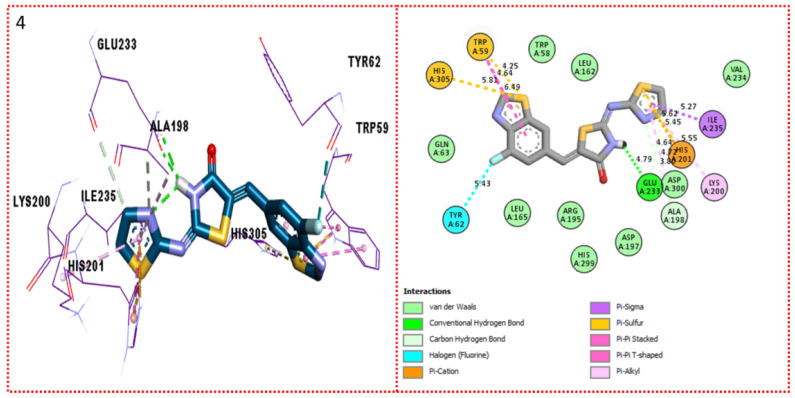
Protein–ligand interaction profile for active analogue against α-amylase represents the surface of the corresponding enzyme and indicates the PLI profile for compound **4**.

**Figure 8:**
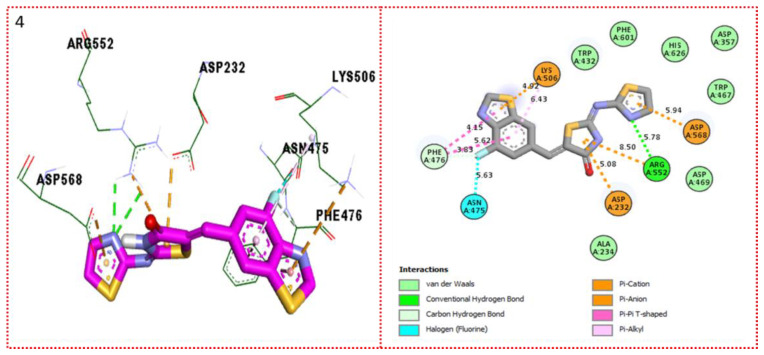
Protein–ligand interaction profile for active analogue against α-glucosidase represents the surface of the corresponding enzyme and indicates the PLI profile for compound **4**.

**Figure 9:**
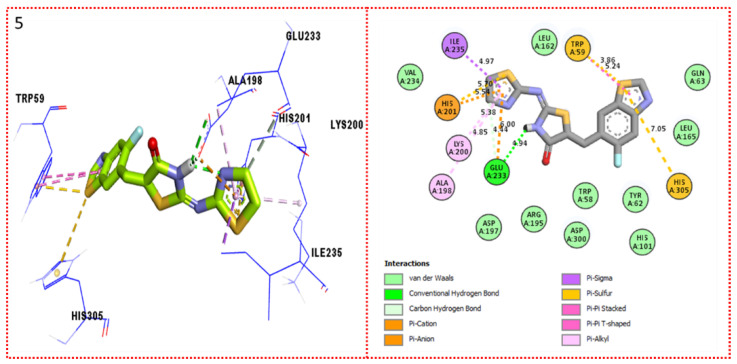
Protein–ligand interaction profile for active analogue against α-amylase represents the surface of the corresponding enzyme and indicates the PLI profile for compound **5**.

**Figure 10:**
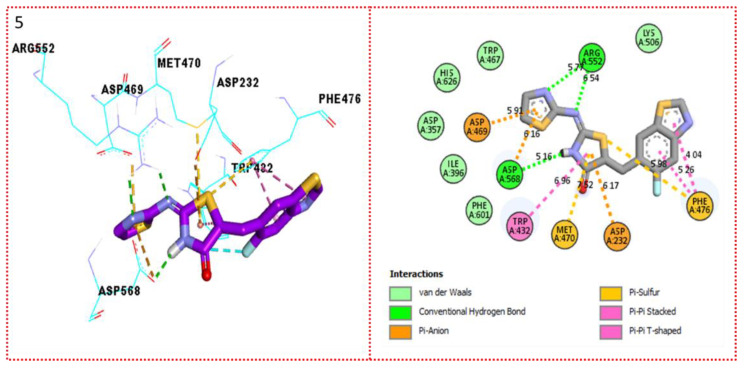
Protein–ligand interaction profile for active analogue against α-amylase represents the surface of the corresponding enzyme and indicates the PLI profile for compound **5**.

**Figure 11:**
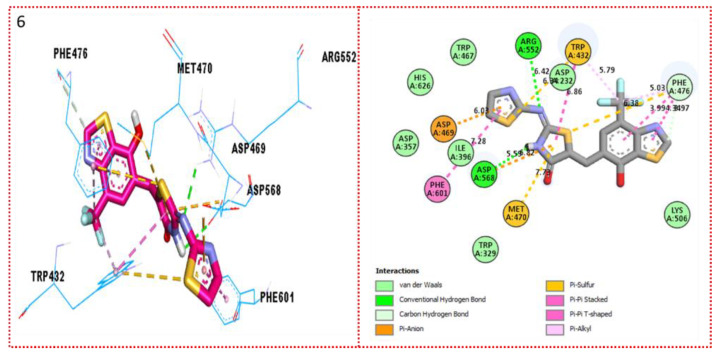
Protein–ligand interaction profile for active analogue against α-amylase represents the surface of the corresponding enzyme and indicates the PLI profile for compound **6**.

**Figure 12:**
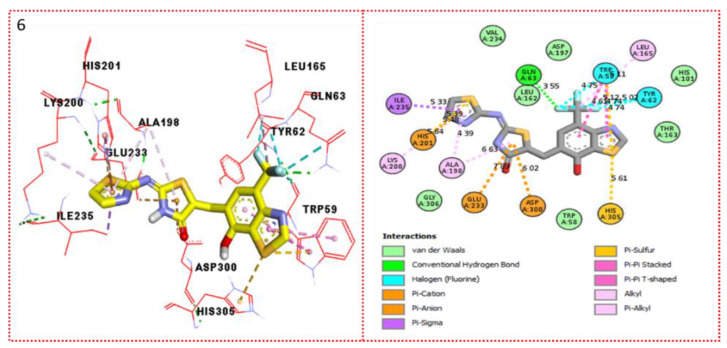
Protein–ligand interaction profile for active analogue against α-glucosidase represents the surface of the corresponding enzyme and indicates the PLI profile for compound **6**.

A molecular docking study revealed the interactive residues with active sites of ligands. The interactions are generally obtained between a protein and the ligand of interest. The target enzymes such as α-amylase and α-glucosidase were obtained from the online source www.rcsb.org, accessed on 14 September 2022. The better interaction of ligand molecules against targeted enzymes might be due to the presence of attached substituents. Here, the nature of substituents is much more important for binding interactions. Thus, the electron-donating group activates the ring by a negative charge, which dominantly increases the interaction toward the positive center or deficient species. In this study, analogue **4** bore fluoro at the *meta-*position, being an electron-withdrawing property, but a strong hydrogen bond was found with better interactions, followed by the interaction procedure of analogue **5**, which bore flouro at the *ortho-*position, showing a somewhat greater interactive property that might be due to the presence of a flouro group at the *ortho-*position; this increases the chances of hydrogen bonding, while analogue **6** has a trifluoro group along with a hydroxyl group, exhibiting excellent interactions, and their superposed surface complex structure is illustrated in Figure 7, Figure 8, Figure 9, Figure 10, Figure 11 and Figure 12. In addition, these ligands (**4**, **5**, and **6**) were found with varied binding modalities against targeted enzymes, which demonstrated excellent potency in an in vitro study and were found with the best potential (in silico). The best mode of analogues **5** and **6** was observed when compared with acarbose, and the binding affinity of derivatives against α-amylase and α-glucosidase and the RMSD value were found to be lower than that of the standard drug, as shown in Table 2, Table 3, Table 4 and Table 5.

In the case of the flouro-substituted derivative **4**, its protein–ligand interaction profile exhibited varied interactive residues for α-amylase with a distance of 3.16–6.49 A, as shown in Figure 6, and the interaction with different amino acids such as HIS-A-305(π-S), TRP-A-59 (π-S), ILE-A-235(π-σ), HIS-A-201(π-cation), LYS-A-200(π-R), GLU-A-233(H-B), and TYR-A-62(H-F), as shown in Figure 7.

Similarly, for α-glucosidase, the interactive residues with a distance of 3.89–8.50 A are LYS-A-506(π-anion), LYS-A-506(π-R), ASP-A-568(π-cation), ARG-A-552(H-B), ASP-A-232(π-anion), ASN-A-475(H-F), and PHE-A-476(C-H), as shown in Figure 8.

In the case of fluoro-substituted analogue **5** against α-amylase, the interactive residues in the range from 3.86–7.05 A, are ILE-A-235(π-σ), TRP-A-59(π-S), HIS-A-305(π-S), GLU-A-233(H-B), GLU-A-233(π-cation), ALA-A-198(π-R), LYS-A-2-00(π-R), and HIS-A-201(π-anion), as shown in Figure 9.

Similarly, for α-glucosidase, the interactive residues with a distance of 4.04–7.52 A are ARG-A-552(H-B), PHE-A-476(π-S), PHE-A-476(π-π-stacked), ASP-A-232(π-anion), MET-A-470 (π-S), TRP-A-432(π-π-T-shaped), ASP-A-568(H-B), and ASP-A-469(π-anion), as shown in Figure 10.

In the case of triflouro analogue **6**, the interactive residues against α-amylase in the range from 3.99–7.72 A are ARG-A-552(H-B), TRP-A-432(π-S), TRP-A-432(π-π-stacked), PHE-A-(C-H), PHE-A-(π-π-T-shaped), ME-A-470(π-S), ASP-A-568(H-B), ASP-A-658(π-anion), ILE-A-376(C-H), PHE-A-601(π-π-T-shaped), ASP-A-469(π-anion), etc., as shown in Figure 11.

Similarly, for α-glucosidase, the interactive residues with a distance of 3.33–7.89 A are GLN-A-63(H-B), TRP-A-55(H-F), TRP-A-55(π-π-T-shaped), LEU-A-165(π-R), TYR-A-62(H-F), HIS-A-305(π-S), ASP-A-300(π-anion), GLU-A-233(π-cation), ALA-A-158(π-R), ALA-A-198(R), HIS-A-201(π-anion), LYS-A-200(π-R), and ILE-A-235(π-σ), as shown in Figure 12.

**Figure 13:**
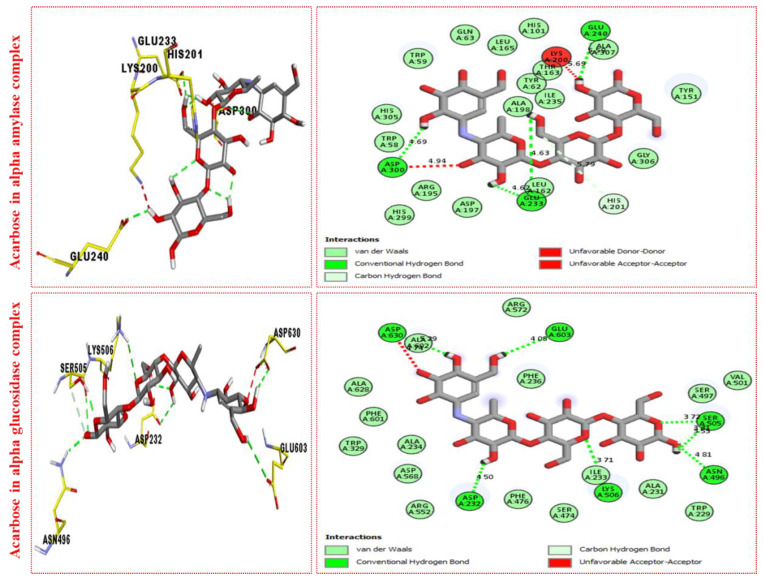
The PLI profile for potent compounds against α-amylase and α-glucosidase represents the surface of the corresponding enzyme and indicates the PLI profile for acarbose.

The comparison study with the standard drug acarbose exhibited the following interactions against the α-amylase enzyme with a distance of 4.62–5.79 A and their interactive residues such as ASP-A-300(H-B), ASP-A-300(Donor-donor), GLU-A-233 (H-B). LEU-A-162(VW), HIS-A-201(C-H), ALA-A307 (VW), GLU-A-240(H-B), LYS-A-200(Acceptor-acceptor), etc. Similarly, α-glucosidase showed a varied range of distance from 3.71–5.29A and their interactive residues such as ASP-A-630(H-B), ALA-A-492(VW), GLU-A-603(H-B), SER-A-505(H-B), ASN-A-496(H-B), ILE-A-233(VW), ASP-A-232(H-B), etc., as illustrated in Figure 13. Most of the synthesized ligands showed better interaction when compared with the standard drug acarbose, due to an attached functional group.

It was concluded that among the tested compounds, the potent behavior was shown by analogues **4** and **5**; both were found with excellent potential against the targeted enzymes. In the docked profile, when compared with the standard drug acarbose, both analogs were found to have excellent interactions. This might be due to the presence of electrons withdrawing and donating groups, respectively, which increased the binding modalities with different interactions such as pi–anion, pi–cation, pi–sulfur, hydrogen bonding, and pi–pi. The interactive ability of these derivatives is due to the presence of benzene rings and different heteroatoms.

## 3. Materials and Methods

### 3.1. General Information 

For the synthesis of thiazolidinone-based benzothiazole derivatives, all required reagent reagents and related chemicals were obtained from Sigma Aldrich USA. The reaction progress was monitored at every step by using thin layer chromatography (TLC) containing aluminum plate, which was covered by precoated silica gel and was purchased from Kieselgel 60,254, E. Merck, Germany. Moreover, visualization of chromatograms was done via UV at 254 and 365 nm. Furthermore, spectroscopic characterization such as HREI-MS and NMR were done by using varied spectrometers, Finnigan MAT-311A and Advance Bruker AM 600 MHz machines, respectively.

### 3.2. General Procedure for the Synthesis of Thiazole-Based Thiazolidinone Derivatives **1**–**15**

An equimolar mixture of 2-aminothiazole **I** (2.0 mmol) and ammonium isothiocyanate (2.0 mmol) were dissolved in methanol (25 mL) and acetic acid (3 drops) were added as catalyst and the reaction mixture was refluxed for about 3 h, yielding thiazole-based thiourea analogue **II**, which was then refluxed for about 4 h in the acetic medium (20 mL) along with chloroacetic acid (2.0 mmol) and afforded thiazole-based thiazolidinedione derivative **III**. In the last step, thiazolidinone-based benzothiazole derivatives **1**–**15** were obtained by mixing thiazole-based thiazolidinone derivative **III** with different substituted benzothiazole-bearing aldehyde groups (1.0 mmol) in methanol (20 mL) in the presence of K_2_CO_3_ (few drops of 0.2 M) under refluxed condition for about 5 h. After completion of reactions, the products were washed with *n-*hexane in order to remove impurities and pure products were collected by means of filtrations. All reaction progress was monitored by mean of thin layer chromatography (TLC), which clearly indicates the difference between the reactant and products spots. Furthermore, spectroscopic characterizations were determined through NMR and HREI-MS. 

### 3.3. Spectral Analysis

Spectral analysis is provided in the supplementary data.

## 4. Conclusions

Thiazolidinone-based benzothiazole derivatives **1**–**15** were synthesized through a series of reactions and evaluated against α-amylase and α-glucosidase enzymes. All the synthesized derivatives displayed good to moderate inhibitory activity, but derivative **4** and **5** were found with excellent activities in both cases, when compared with the standard drug acarbose. A molecular docking study also revealed the significant binding modalities of subjected candidates. These candidates’ interactive properties were also compared, by employing a molecular docking study with the standard drug acarbose. Analogues **4** and **5** were found with varied interactions at different ranges, which indicate the active site in the molecule as well as in the enzymes. This study identifies a new class of thiazolidinone-based benzothiazole derivatives as α-amylase and α-glucosidase inhibitors.

## Figures and Tables

**Table 1:** Thiazolidinedione-based benzothiazole derivatives **1**–**15** with biological inhibitory profile.

Synthesized Compounds	R	α-Amylase IC_50_ (µM)	α-Glucosidase IC_50_ (µM)
**1**	6-Nitro	7.70 ± 0.20	8.80 ± 0.20
**2**	7-Nitro	6.60 ± 0.30	7.20 ± 0.30
**3**	7-Chloro-6-nitro	9.90 ± 0.40	11.70 ± 0.20
**4**	7-Fluoro	2.40 ± 0.70	3.50 ± 0.70
**5**	7-Fluoro	2.30 ± 0.05	4.80 ± 0.10
**6**	7-Trifluoromethyl-4-hydroxy	2.10 ± 0.70	3.20 ± 0.70
**7**	4,7-dihydroxy	3.90 ± 0.80	4.50 ± 0.80
**8**	4-hydroxy-6-methoxy	11.60 ± 0.60	12.70 ± 0.80
**9**	7-(2-chloro-3-nitrophenyl)	28.50 ± 0.30	29.20 ± 0.40
**10**	4-dimethylamino	31.20 ± 0.10	33.60 ± 0.20
**11**	4-Chloro-6-hydroxy	7.90 ± 0.10	9.85 ± 0.20
**12**	4-Chloro-7-hydroxy	10.30 ± 0.20	11.10 ± 0.30
**13**	4-Chloro	9.70 ± 0.20	10.50 ± 0.20
**14**	6,7-dichloro	13.20 ± 0.30	13.80 ± 0.40
**15**	7-(3-cyanophenyl)	37.50 ± 0.70	39.40 ± 0.80
**Standard drug acarbose**		9.10 ± 0.10	10.70 ± 0.10

**Table 2:** The binding affinity and RMSD value of derivative **4** against α-amylase and α-glucosidase.

Derivative-**4** against α-amylase	**Mode**	**Affinity Kcal/mol**	**Dist from rmsdl.b**	**Best Mode rmsdu.b**
	1	−8.7	0.000	0.000
	2	−8.2	3.509	9.326
	3	−8.1	3.524	9.035
	4	−7.7	2.064	2.537
	5	−7.7	3.222	4.078
	6	−7.5	2.491	3.578
	7	−7.4	3.997	9.249
	8	−7.2	3.265	4.589
	9	−7.2	4.618	6.871
Derivative-**4** against α-glucosidase	1	−7.2	0.000	0.000
	2	−7.1	4.386	8.557
	3	−7.1	2.493	2.825
	4	−7.1	5.907	8.803
	5	−6.5	4.612	5.490
	6	−6.2	3.062	8.751
	7	−6.1	4.812	8.361
	8	−5.9	4.444	8.781
	9	−5.9	6.901	8.970

**Table 3:** The binding affinity and RMSD value of derivative-**5** against α-amylase and α-glucosidase.

Derivative-**5** against α-amylase	**Mode**	**Affinity Kcal/mol**	**Dist from rmsdl.b**	**Best Mode rmsdu.b**
	1	−8.1	0.000	0.000
	2	−7.7	2.690	3.719
	3	−7.7	4.164	9.216
	4	−7.3	1.867	2.573
	5	−7.2	3.246	5.204
	6	−7.2	3.340	9.046
	7	−7.1	2.511	8.599
	8	−6.6	12.800	14.844
	9	−6.5	2.796	8.422
Derivative-**5** against α-glucosidase	1	−7.3	0.000	0.000
	2	−7.2	4.474	8.247
	3	−7.0	2.535	3.033
	4	−6.5	6.261	8.114
	5	−6.3	5.148	5.843
	6	−6.2	3.705	4.420
	7	−6.0	6.046	9.057
	8	−6.0	16.081	17.997
	9	−5.9	6.508	9.537

**Table 4:** The binding affinity and RMSD value of derivative-**6** against α-amylase and α-glucosidase.

Derivative-**6** against α-amylase	**Mode**	**Affinity Kcal/mol**	**Dist from rmsdl.b**	**Best Mode rmsdu.b**
	1	−8.6	0.000	0.000
	2	−8.1	3.324	8.460
	3	−8.0	3.376	8.475
	4	−7.6	4.032	8.861
	5	−7.5	2.904	8.532
	6	−7.5	3.546	4.849
	7	−7.3	3.664	5.155
	8	−7.3	4.675	9.102
	9	−7.3	3.260	3.961
Derivative-**6** against α-glucosidase	1	−7.3	0.000	0.000
	2	−6.8	3.771	4.374
	3	−6.6	5.743	8.923
	4	−6.4	7.483	8.685
	5	−6.1	5.822	8.279
	6	−6.1	6.616	9.305
	7	−6.0	15.492	16.488
	8	−5.9	13.940	18.195
	9	−5.9	6.522	10.003

**Table 5:** The binding affinity and RMSD value of acarbose against α-amylase and α-glucosidase.

Acarbose against α-amylase	**Mode**	**Affinity Kcal/mol**	**Dist from rmsdl.b**	**Best Mode rmsdu.b**
	1	−8.0	0.000	0.000
	2	−7.8	3.267	11.500
	3	−7.8	3.228	10.552
	4	−7.7	2.163	9.832
	5	−7.5	4.169	7.220
	6	−7.5	5.320	9.443
	7	−7.4	4.477	8.490
	8	−7.3	3.580	11.779
	9	−7.5	5.259	9.041
Acarbose against α-glucosidase	1	−7.3	0.000	0.000
	2	−7.3	3.562	11.099
	3	−7.3	1.489	2.269
	4	−6.9	3817	10.685
	5	−6.9	3,838	10.852
	6	−6.7	7.042	13.565
	7	−6.7	6.866	11.671
	8	−6.6	2.089	11.269
	9	−6.6	4.540	11.478

## Data Availability

Data are contained within the article and supplementary material.

## Supplementary Materials

The following supporting information can be downloaded at: https://www.mdpi.com/article/10.3390/ph15101164/s1. Figure S1. High resolution 13C NMR spectra of (2Z,5Z)-5-((7-hydroxy-4-(trifluoromethyl)benzo[d]thiazol-6-yl)methylene)-2-(thiazol-2-ylimino)thiazolidin-4-one (6); Figure S2. Low resolution Proton NMR Spectra of (2Z,5Z)-5-((7-(2-chloro-3-nitrophenyl)benzo[d] thiazol-6-yl)methylene)-2-(thiazol-2-ylimino)thiazolidin-4-one (9); Figure S3. High resolution Proton NMR Spectra of (2Z,5Z)-5-((7-(2-chloro-3-nitrophenyl)benzo[d] thiazol-6-yl)methylene)-2-(thiazol-2-ylimino)thiazolidin-4-one (9); Figure S4. Low resolution Proton NMR Spectra of (2Z,5Z)-5-((4-(dimethylamino)benzo[d]thiazol-6-yl)methylene)-2-(thiazol-2-ylimino)thiazolidin-4-one (10); Figure S5. High resolution Proton NMR Spectra of (2Z,5Z)-5-((4-(dimethylamino)benzo[d]thiazol-6-yl)methylene)-2-(thiazol-2-ylimino)thiazolidin-4-one (10); Figure S6. Low resolution Proton NMR Spectra of 3-(6-((Z)-((Z)-4-oxo-2-(thiazol-2-ylimino) thiazolidin-5-ylidene)methyl)benzo[d]thiazol-7-yl)benzonitrile (15); Figure S7. High resolution Proton NMR Spectra of 3-(6-((Z)-((Z)-4-oxo-2-(thiazol-2-ylimino) thiazolidin-5-ylidene)methyl)benzo[d]thiazol-7-yl)benzonitrile (15); Figure S8. High resolution 13C-NMR Spectra of 3-(6-((Z)-((Z)-4-oxo-2-(thiazol-2-ylimino)thiazolidin-5-ylidene)methyl)benzo[d]thiazol-7-yl)benzonitrile (15).
