# Supplementary material for: Design, Synthesis, In Silico Testing, and In Vitro Evaluation of Thiazolidinone-Based Benzothiazole Derivatives as Inhibitors of α-Amylase and α-Glucosidase
Source: Pharmaceuticals (Basel). 2022 Sep 20;15(10):1164. doi: 10.3390/ph15101164 (PMC9610606; doi:10.3390/ph15101164)
Supplement: Supplementary file 1 [file pharmaceuticals-15-01164-s001.zip › pharmaceuticals-1892154-supplementary.pdf]

Supporting Information

Design, Synthesis, in Silico Testing, and in Vitro Evaluation of Thiazolidinone-Based Benzothiazole Derivatives as Inhibitors of α-Amylase and α-Glucosidase

Shoaib Khan [1,*], Shahid Iqbal [2,*], Marwa Khan [1], Wajid Rehman [1], Mazloom Shah [3,*], Rafaqat Hussain [1], Liaqat Rasheed [1], Yousaf Khan [4], Ayed A. Dera [5], Rami Adel Pashameah [6], Eman Alzahrani [7] and Abd-ElAziem Farouk [8]

[1] Department of Chemistry, Hazara University, Mansehra 21120, Pakistan
[2] Department of Chemistry, School of Natural Sciences (SNS), National University of Science and Technology (NUST), H-12, Islamabad 46000, Pakistan
[3] Department of Chemistry, Abbottabad University of Science and Technology (AUST), Abbottabad 22500, Pakistan
[4] Department of Chemistry, COMSATS University Islamabad Campus, Islamabad 45550, Pakistan
[5] Department of Clinical Laboratory Sciences, College of Applied Medical Sciences, King Khalid University, Abha 61421, Saudi Arabia
[6] Department of Chemistry, Faculty of Applied Science, Umm Al-Qura University, Makkah 24230, Saudi Arabia
[7] Department of Chemistry, College of Science, Taif University, P.O. Box 11099, Taif 21944, Saudi Arabia
[8] Department of Biotechnology, College of Science, Taif University, P.O. Box 11099, Taif 21944, Saudi Arabia
* Correspondence: shoaibkhanswati@gmail.com (S.K.); shahidgcs10@yahoo.com (S.I.); shahmazloom@yahoo.com (M.S.)

5.2. Spectral analysis

5.2.1. (2Z,5Z)-5-((5-nitrobenzo[d]thiazol-6-yl)methylene)-2-(thiazol-2-ylimino)thiazolidin-4-one (1)

Yield 82%, m.p. 235–236 °C, Light yellow. ^{1}H-NMR (600 MHz, DMSO-*d6*): δ 11.69 (s, 1H, NH), 9.37 (s, 1H, Benzo-thiazole-H), 8.34 (s, 1H, Benzo-thiazole-H), 8.26 (s, 1H, Benzo-thiazole-H), 7.33 (s, 1H, C-H), 7.13 (d, *J* = 8.0Hz, 1H, Thiazole-H), 6.84 (d, *J* = 7.8Hz, 1H, Thiazole-H), ^{13}C-NMR (150 MHz, DMSO-*d6*):δ 162.2, 149.3, 148.4, 148.8, 127.4, 125.0, 123.4, 122.2, 121.1, 119.7, 118.8, 115.6, 109.0, 100.5. HR EI-MS: *m/z*calcd for $C_{14}H_7N_5O_3S_3$ [M]$^+$ 388.8162; Found: 388.3820.

5.2.2. (2Z,5Z)-5-((4-nitrobenzo[d]thiazol-6-yl)methylene)-2-(thiazol-2-ylimino)thiazolidin-4-one (2)

Yield 80%, m.p. 233–234ºC, Yellow. ^{1}H-NMR (600 MHz, DMSO-*d*$_6$): *δ* 11.70 (s, 1H, NH), 9.38 (s, 1H, Benzo-thiazole-H), 8.35 (s, 1H, Benzo-thiazole-H), 8.27 (s, 1H, Benzo-thiazole-H), 7.34 (s, 1H, C-H), 7.14 (d, *J* = 8.2Hz, 1H, Thiazole-H), 6.85 (d, *J* = 7.4Hz, 1H, Thiazole-H), ^{13}C-NMR (150 MHz, DMSO-*d*$_6$):*δ* 162.3, 149.4, 148.5, 148.9, 127.6, 125.3, 123.2, 122.7, 121.3, 119.8, 118.6, 115.3, 109.9, 100.6. HR EI-MS: *m/z*calcd for $C_{14}H_7N_5O_3S_3$ [M]$^+$ 388.8159; Found: 388.3816.

5.2.3. (2Z,5Z)-5-((4-chloro-5-nitrobenzo[d]thiazol-6-yl)methylene)-2-(thiazol-2-ylimino)thiazolidin-4-one (3)

Yield 81%, m.p. 233–234ºC, Light yellow. ^{1}H-NMR (600 MHz, DMSO-*d*$_6$): *δ* 11.62 (s, 1H, NH), 9.39 (s, 1H, Benzo-thiazole-H), 8.36 (s, 1H, Benzo-thiazole-H), 7.33 (s, 1H, C-H), 7.10 (d, *J* = 8.1Hz, 1H, Thiazole-H), 6.86 (d, *J* = 8.0Hz, 1H, Thiazole-H), ^{13}C-NMR (150 MHz, DMSO-*d*$_6$):*δ* 162.5, 149.6, 148.9, 148.0, 127.6, 125.9, 123.5, 122.8, 121.5, 119.4, 118.6, 115.5, 109.4, 100.9. HR EI-MS: *m/z*calcd for $C_{14}H_6ClN_5O_3S_3$ [M]$^+$ 422.9130; Found: 422.8980.

5.2.4. (2Z,5Z)-5-((4-fluorobenzo[d]thiazol-6-yl)methylene)-2-(thiazol-2-ylimino)thiazolidin-4-one (4)

Yield 86%, m.p. 237–238 ºC, Light green. ^{1}H-NMR (600 MHz, DMSO-*d*$_6$): *δ* 11.92 (s, 1H, NH), 10.45 (s, 1H, Benzo-thiazole-H), 8.88 (s, 1H, Benzo-thiazole-H), 8.81 (s, 1H, Benzo-thiazole-H), 8.57 (s, 1H, C-H), 7.59 (d, *J* = 6.7Hz, 1H, Thiazole-H), 6.96 (d, *J* = 7.4Hz, 1H, Thiazole-H), ^{13}C-NMR (150 MHz, DMSO-*d*$_6$):*δ* 159.6, 149.6, 147.3, 142.7, 135.8, 129.8, 127.6, 121.2, 119.9, 118.7, 116.0, 114.6, 111.3, 100.9. HR EI-MS: *m/z*calcd for $C_{14}H_7FN_4OS_3$ [M]$^+$ 361.9629; Found: 3651.9254.

5.2.5. (2Z,5Z)-5-((5-fluorobenzo[d]thiazol-6-yl)methylene)-2-(thiazol-2-ylimino)thiazolidin-4-one (5)

Yield 82%, m.p. 233–234 ºC, Light green. ^{1}H-NMR (600 MHz, DMSO-*d6*): *δ* 11.90 (s, 1H, NH), 10.41 (s, 1H, Benzo-thiazole-H), 8.84 (s, 1H, Benzo-thiazole-H), 8.79 (s, 1H, Benzo-thiazole-H), 8.55 (s, 1H, C-H), 7.56 (d, *J* = 7.0Hz, 1H, Thiazole-H), 6.94 (d, *J* = 7.1Hz, 1H, Thiazole-H), ^{13}C-NMR (150 MHz, DMSO-*d6*):*δ* 159.5, 149.5, 147.2, 142.6, 135.7, 129.7, 127.5, 121.1, 119.8, 118.6, 116.1, 114.5, 111.2, 100.8. HR EI-MS: *m/z* calcd for $C_{14}H_7FN_4OS_3$ [M]$^+$ 361.9521; Found: 3651.9134.

5.2.6. (2Z,5Z)-5-((7-hydroxy-4-(trifluoromethyl)benzo[d]thiazol-6-yl)methylene)-2-(thiazol-2-ylimino)thiazolidin-4-one (6)

Yield 85%, m.p. 232–233ºC, Light yellow. ^{1}H-NMR (600 MHz, DMSO-*d6*): *δ* 11.70 (s, 1H, NH), 11.33 (s, 1H, OH), 9.70 (s, 1H, Benzo-thiazole-H), 8.42 (s, 1H, Benzo-thiazole-H), 7.88 (s, 1H, C-H), 7.60 (d, *J* = 6.7Hz, 1H, Thiazole-H), 6.85 (d, *J* = 8.3Hz, 1H, Thiazole-H), ^{13}C-NMR (150 MHz, DMSO-*d6*):*δ* 168.4, 167.1, 148.7, 137.6, 136.4, 132.9, 130.1, 129.6, 127.5, 126.7, 125.0, 120.6, 118.5, 116.0, 112.1. HR EI-MS: *m/z* calcd for $C_{15}H_7F_3N_4O_2S_3$ [M]$^+$ 427.8691; Found: 427.8630.

5.2.7. (2Z,5Z)-5-((4,7-dihydroxybenzo[d]thiazol-6-yl)methylene)-2-(thiazol-2-ylimino)thiazolidin-4-one (7)

Yield 92%, m.p. 232–233ºC, Light brown. ^{1}H-NMR (600 MHz, DMSO-*d6*): *δ* 11.73 (s, 1H, NH), 11.38 (s, 1H, OH), 11.32 (s, 1H, OH), 9.73 (s, 1H, Benzo-thiazole-H), 8.49 (s, 1H, Benzo-thiazole-H), 7.70 (s, 1H, C-H), 7.63 (d, *J* = 7.7Hz, 1H, Thiazole-H), 6.89 (d, *J* = 7.3Hz, 1H, Thiazole-H), ^{13}C-NMR (150 MHz, DMSO-*d6*):*δ* 160.9, 148.1, 129.6, 129.3, 128.2, 126.7, 125.5, 124.8, 122.4, 119.5, 116.1, 115.0, 112.2, 101.8. HR EI-MS: *m/z*calcd for $C_{14}H_8N_4O_3S_3$ [M]$^+$ 375.9429; Found: 375.9380.

5.2.8. (2Z,5Z)-5-((7-hydroxy-5-methoxybenzo[d]thiazol-6-yl)methylene)-2-(thiazol-2-ylimino)thiazolidin-4-one (8)

Yield 84%, m.p. 227–228ºC, Light gray. ^{1}H-NMR (600 MHz, DMSO-*d₆*): *δ* 11.69 (s, 1H, NH), 11.26 (s, 1H, OH), 9.66 (s, 1H, Benzo-thiazole-H), 8.38 (s, 1H, Benzo-thiazole-H), 7.61 (s, 1H, C-H), 7.58 (d, *J* = 7.6Hz, 1H, Thiazole-H), 6.77 (d, *J* = 7.8Hz, 1H, Thiazole-H), 2.41 (s, 3H, CH_3), ^{13}C-NMR (150 MHz, DMSO-d_6):*δ* 160.7, 148.9, 129.2, 129.8, 128.3, 126.4, 125.0, 124.2, 122.1, 119.7, 116.6, 115.5, 112.9, 101.4, 43.6. HR EI-MS: *m/z*calcd for $C_{15}H_{10}N_4O_3S_3$ $[M]^+$ 389.4511; Found: 389.4480.

5.2.9. (2Z,5Z)-5-((7-(2-chloro-3-nitrophenyl)benzo[d]thiazol-6-yl)methylene)-2-(thiazol-2-ylimino)thiazolidin-4-one (9)

Yield 81%, m.p. 241–242 ºC, Light brown. ^{1}H-NMR (600 MHz, DMSO-d_6): *δ* 11.38 (s, 1H, NH), 8.66 (s, 1H, Benzo-thiazole-H), 8.45 (d, *J* = 7.9Hz, 1H, Benzo-thiazole-H), 8.26 (s, 1H, C-H), 8.17 (t, *J* = 8.1Hz, 1H, Ar-H), 7.80 (d, *J* = 7.9Hz, 1H, Benzo-thiazole-H), 7.65 (d, *J* = 7.9Hz, 1H, Ar-H), 7.57 (d, *J* = 7.9Hz, 1H, Ar-H), 7.05 (d, *J* = 8.2Hz, 1H, Thiazole-H), 7.00 (d, *J* = 8.6Hz, 1H, Thiazole-H),^{13}C-NMR (150 MHz, DMSO-d_6):*δ* 165.5, 163.6, 162.4, 149.0, 147.6, 142.4, 140.1, 138.0, 134.8, 131.7, 130.0, 128.2, 127.5, 126.9, 124.0, 123.7, 120.1, 117.7, 115.0. HR EI-MS: *m/z* calcd for $C_{20}H_{10}ClN_5O_3S_3$ $[M]^+$ 498.0937; Found: 498.0830.

5.2.10. (2Z,5Z)-5-((4-(dimethylamino)benzo[d]thiazol-6-yl)methylene)-2-(thiazol-2-ylimino)thiazolidin-4-one (10)

Yield 84%, m.p. 227–228 ºC, Light gray. ^{1}H-NMR (600 MHz, DMSO-d_6): *δ* 11.36 (s, 1H, NH), 8.18 (d, *J* = 2.2Hz, 1H, Benzo-thiazole-H), 8.12 (d, *J* = 2.4Hz, 1H, Benzo-thiazole-H), 7.87 (s, 1H, Benzo-thiazole-H), 7.24 (s, 1H, C-H), 7.58 (d, *J* = 7.7Hz, 1H, Thiazole-H), 7.20 (d, *J* = 7.5Hz, 1H, Thiazole-H), 2.38 (s, 6H, -NCH3), ^{13}C-NMR (150 MHz, DMSO-d_6):*δ* 160.4, 159.3, 149.9, 147.7,

142.8, 135.3, 129.1, 127.4, 121.6, 119.7, 118.0, 116.8, 114.6, 111.7, 39.6, 39.5. HR EI-MS: *m/z*calcd for $C_{16}H_{13}N_5OS_3$ $[M]^+$ 387.0630; Found: 387.0518.

5.2.11. (2Z,5Z)-5-((7-chloro-5-hydroxybenzo[d]thiazol-6-yl)methylene)-2-(thiazol-2-ylimino)thiazolidin-4-one (11)

Yield 90%, m.p. 236–237°C, Light yellow. ^{1}H-NMR (600 MHz, DMSO-*d6*): δ 11.86 (s, 1H, NH), 11.59 (s, 1H, OH), 9.80 (s, 1H, Benzo-thiazole-H), 8.36 (s, 1H, Benzo-thiazole-H), 7.51 (s, 1H, C-H), 7.56 (d, J = 6.9Hz, 1H, Thiazole-H), 6.85 (d, J = 8.0Hz, 1H, Thiazole-H), ^{13}C-NMR (150 MHz, DMSO-*d6*):δ 159.3, 147.8, 128.7, 128.6, 127.5, 125.4, 124.6, 123.9, 121.5, 118.6, 115.7, 114.7, 111.4, 100.9. HR EI-MS: *m/z*calcd for $C_{14}H_7ClN_4O_2S_3$ $[M]^+$ 393.9155; Found: 393.9060.

5.2.12. (2Z,5Z)-5-((7-chloro-4-hydroxybenzo[d]thiazol-6-yl)methylene)-2-(thiazol-2-ylimino)thiazolidin-4-one (12)

Yield 83%, m.p. 234–235°C, Yellowish white. ^{1}H-NMR (600 MHz, DMSO-*d6*): δ 11.88 (s, 1H, NH), 11.61 (s, 1H, OH), 9.82 (s, 1H, Benzo-thiazole-H), 8.38 (s, 1H, Benzo-thiazole-H), 7.54 (s, 1H, C-H), 7.59 (d, J = 7.9Hz, 1H, Thiazole-H), 6.87 (d, J = 7.0Hz, 1H, Thiazole-H), ^{13}C-NMR (150 MHz, DMSO-*d6*):δ 159.2, 147.7, 128.6, 128.5, 127.4, 125.3, 124.5, 123.8, 121.4, 118.5, 115.6, 114.6, 111.3, 100.8. HR EI-MS: *m/z*calcd for $C_{14}H_7ClN_4O_2S_3$ $[M]^+$ 393.9130; Found: 393.9003.

5.2.13. (2Z,5Z)-5-((7-chlorobenzo[d]thiazol-6-yl)methylene)-2-(thiazol-2-ylimino)thiazolidin-4-one (13)

Yield 89%, m.p. 226–227°C, Light yellow. ^{1}H-NMR (600 MHz, DMSO-*d6*): δ 11.70 (s, 1H, NH), 9.22 (s, 1H, Benzo-thiazole-H), 8.55 (s, 1H, Benzo-thiazole-H), 8.44 (s, 1H, Benzo-thiazole-H), 8.37 (s, 1H, C-H), 7.50 (d, J = 7.2Hz, 1H, Thiazole-H), 6.80 (d, J = 7.8Hz, 1H, Thiazole-H), ^{13}C-NMR (150 MHz, DMSO-*d6*):δ 159.6, 149.6, 147.3, 142.7, 135.8, 129.8, 127.6, 121.2, 119.9, 118.7,

116.0, 114.6, 111.3, 101.9. HR EI-MS: *m/z*calcd for $C_{14}H_7ClN_4OS_3$ $[M]^+$ 377.7210; Found: 377.7180.

5.2.14. (2Z,5Z)-5-((4,5-dichlorobenzo[d]thiazol-6-yl)methylene)-2-(thiazol-2-ylimino)thiazolidin-4-one (14)

Yield 84%, m.p. 235–236ºC, Light yellow. ^{1}H-NMR (600 MHz, DMSO-d_6): δ 11.66 (s, 1H, NH), 9.17 (s, 1H, Benzo-thiazole-H), 8.48 (s, 1H, Benzo-thiazole-H), 8.33 (s, 1H, C-H), 7.45 (d, J = 7.1Hz, 1H, Thiazole-H), 6.77 (d, J = 6.8Hz, 1H, Thiazole-H), ^{13}C-NMR (150 MHz, DMSO-d_6):δ 159.8, 149.4, 147.0, 142.3, 135.2, 129.7, 127.5, 121.1, 119.8, 118.6, 116.7, 114.4, 111.2, 101.9. HR EI-MS: *m/z*calcd for $C_{14}H_6Cl_2N_4OS_3$ $[M]^+$ 411.8238; Found: 411.8028.

5.2.15. 3-(6-((Z)-((Z)-4-oxo-2-(thiazol-2-ylimino)thiazolidin-5-ylidene)methyl)benzo[d]thiazol-7-yl)benzonitrile (15)

Yield 88%, m.p. 243–244 ºC, Light brown. ^{1}H-NMR (600 MHz, DMSO-d_6): δ 11.33 (s, 1H, NH), 9.68 (s, 1H, Benzo-thiazole-H), 8.33 (d, J = 6.7Hz, 1H, Benzo-thiazole-H), 8.14 (d, J = 6.7Hz, 1H, Benzo-thiazole-H),8.19-8.15 (m, 1H, Ar-H), 7.88 (s, 1H, C-H),7.26 (d, J = 7.2Hz, 1H, Thiazole-H), 7.17 (dd, J = 8.2, 1.3Hz, 1H, Ar-H), 7.06 (d, J = 8.6Hz, 1H, Thiazole-H),6.97 (t, J = 1.9Hz, 1H, Ar-H), ^{13}C-NMR (150 MHz, DMSO-d_6): δ 168.9, 148.2, 148.1, 146.1, 145.9, 141.5, 135.6, 135.0, 126.0, 124.8, 121.5, 121.0, 120.8, 119.0, 117.8, 114.9, 114.1, 112.3, 111.6, 109.8, 43.3. HR EI-MS: *m/z* calcd for $C_{21}H_{11}N_5OS_3$ $[M]^+$ 445.0113; Found: 445.0104.

5.3 α-amylase inhibitory assay protocol

20μL of α -amylase solution (0.5 mg/ml) was added to phosphate buffer (pH 6.9, 0.02 M, 200 μL). 250 μL of test samples (10-50 μg/mL) was added, to all the above solutions and kept for incubation for 10 min.1% starch solution (200μL) was further added and again kept for incubation for 10 min at a temperature of 25°C. Termination of the reaction was affected by the addition of 400 μL of 3, 5-dinitrosalicylic acid (DNS) reagent. Lastly, it was incubated in water (at 70°C, 5 min). The

absorbance was recorded using an ELISA microplate reader at 540 nm. Acarbose was served as the standard. All the reactions were carried out in triplicates. The reaction mixture, devoid of the test sample, served as control. The following formula was employed to calculate percentage inhibition. The IC50 values are tabulated in table-7 [1].

% Inhibition = [(Ac – As) /Ac] × 100 Ac-Control absorbance, As-Standard absorbance

5.4 α-glucosidase inhibitory assay protocol

10 µl of the α-glucosidase enzyme, 20 µl of test samples (10-50 µg/ml) and 50 µl, of 0.1M phosphate buffer (pH=6.8), were incubated at 37°C for 15 min in a 96-well plate. 20 µl of p-nitrophenyl—D-glucopyranoside solution was further added as a substrate and again incubated for 20 min at 37 oC. Sodium carbonate (50 µl of 0.1M) was added to terminate the reaction. P-nitrophenol was liberated, during the reaction which was measured at 405nm using an ELISA microplate reader. Acarbose served as the standard. A control was prepared under similar conditions by omitting test samples. All the experiments were carried out in triplicates. The percentage inhibition was calculated by the formula. The IC50 values are tabulated in table-7 [1].

% Inhibition = [(Ac – As) /Ac] × 100 Ac-absorbance for control, As-absorbance for standard.

5.5 Molecular docking assay

In this study the synthesized compounds were analyzed against α-amylase and α-glucosidase enzyme. In the first step protein was prepared by using DSV by removing water molecules and already present ligand were removed save both the target protein as well as prepared ligand in PDB format. The process was further carried out in auto dock in which polar hydrogen and Kollman and Gasteiger charges were added to protein. Selected ligand was also prepared done by using torsion tree to detect root. Moreover, configuration file was generated along with X, Y and Z axis save both ligand and protein in PDBQT format in the same docking folder. At the end command prompt was used to generate varied poses of ligand thus, 9 different poses were obtained in PDBQT format. The dock protein and ligand were then open in DSV to identify the binding interaction of ligand with active sites of enzyme [2].

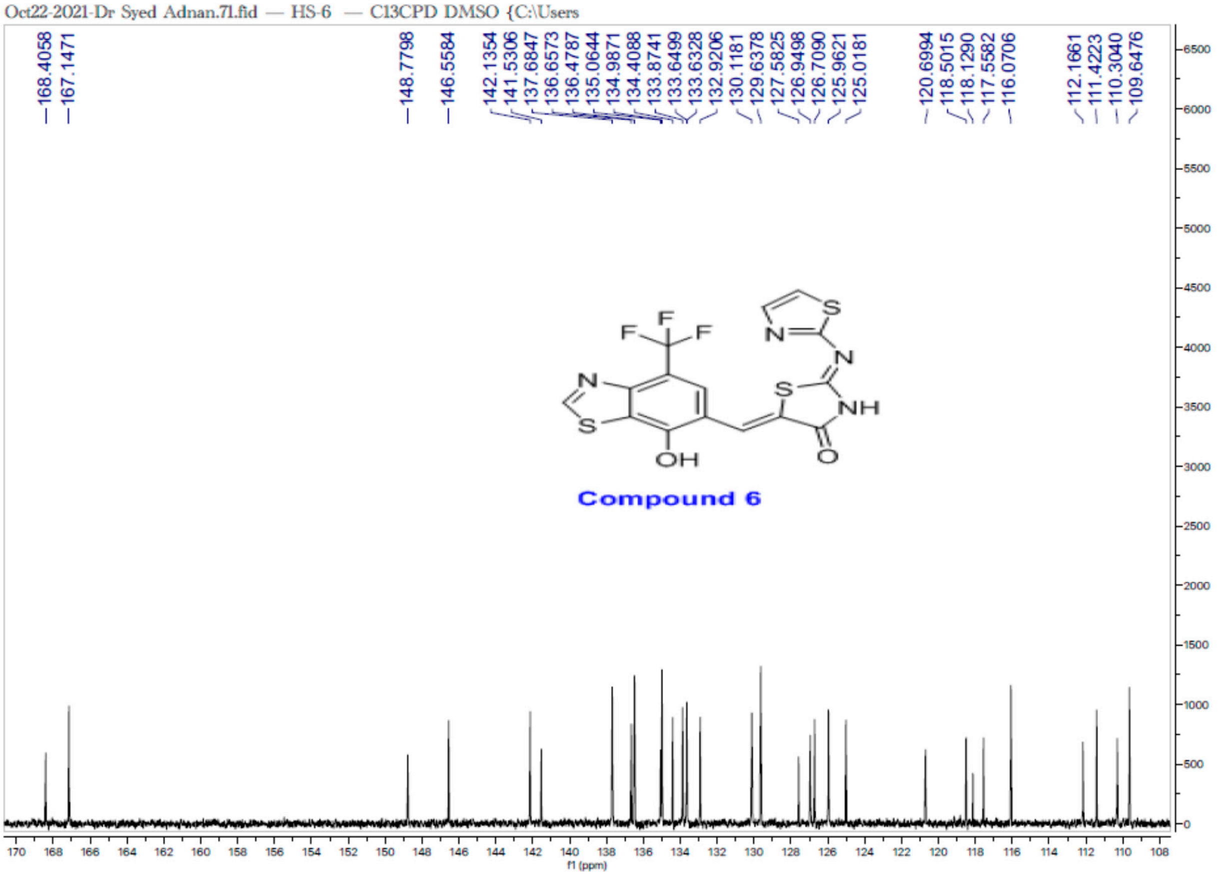

Figure S1. High resolution 13C NMR spectra of (2Z,5Z)-5-((7-hydroxy-4-(trifluoromethyl)benzo[d]thiazol-6-yl)methylene)-2-(thiazol-2-ylimino)thiazolidin-4-one (6)

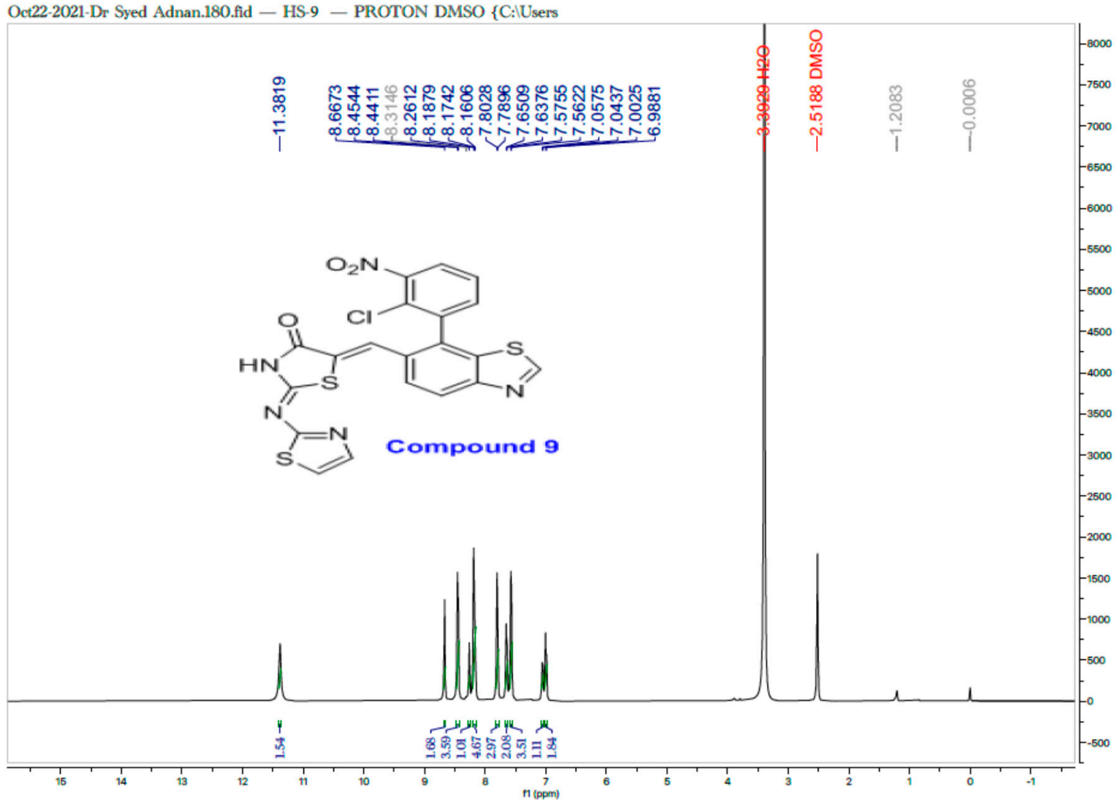
Oct22-2021-Dr Syed Adnan.180.fid — HS-9 — PROTON DMSO {C:\Users
Compound 9
3.3929 H2O
2.5188 DMSO
f1 (ppm)

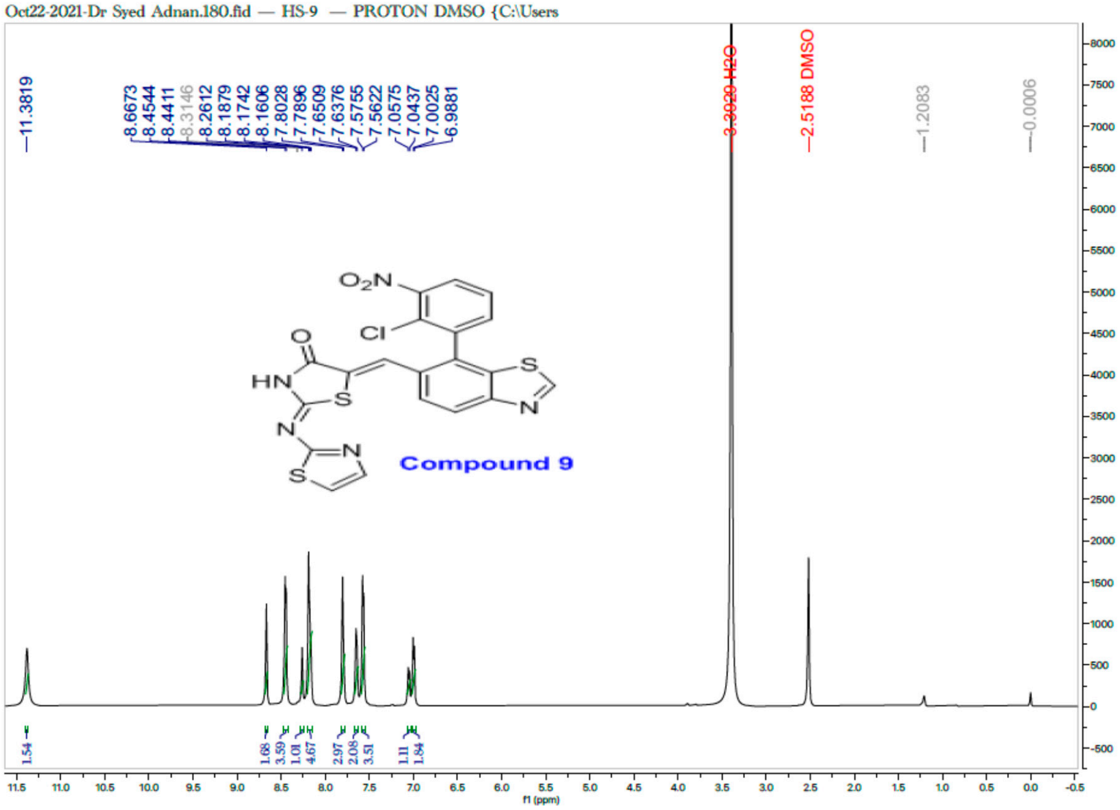
Oct22-2021-Dr Syed Adnan.180.fid — HS-9 — PROTON DMSO {C:\Users
Compound 9
3.3929 H2O
2.5188 DMSO
f1 (ppm)

Figure S2. Low resolution Proton NMR Spectra of (2Z,5Z)-5-((7-(2-chloro-3-nitrophenyl)benzo[d]thiazol-6-yl)methylene)-2-(thiazol-2-ylimino)thiazolidin-4-one (9)

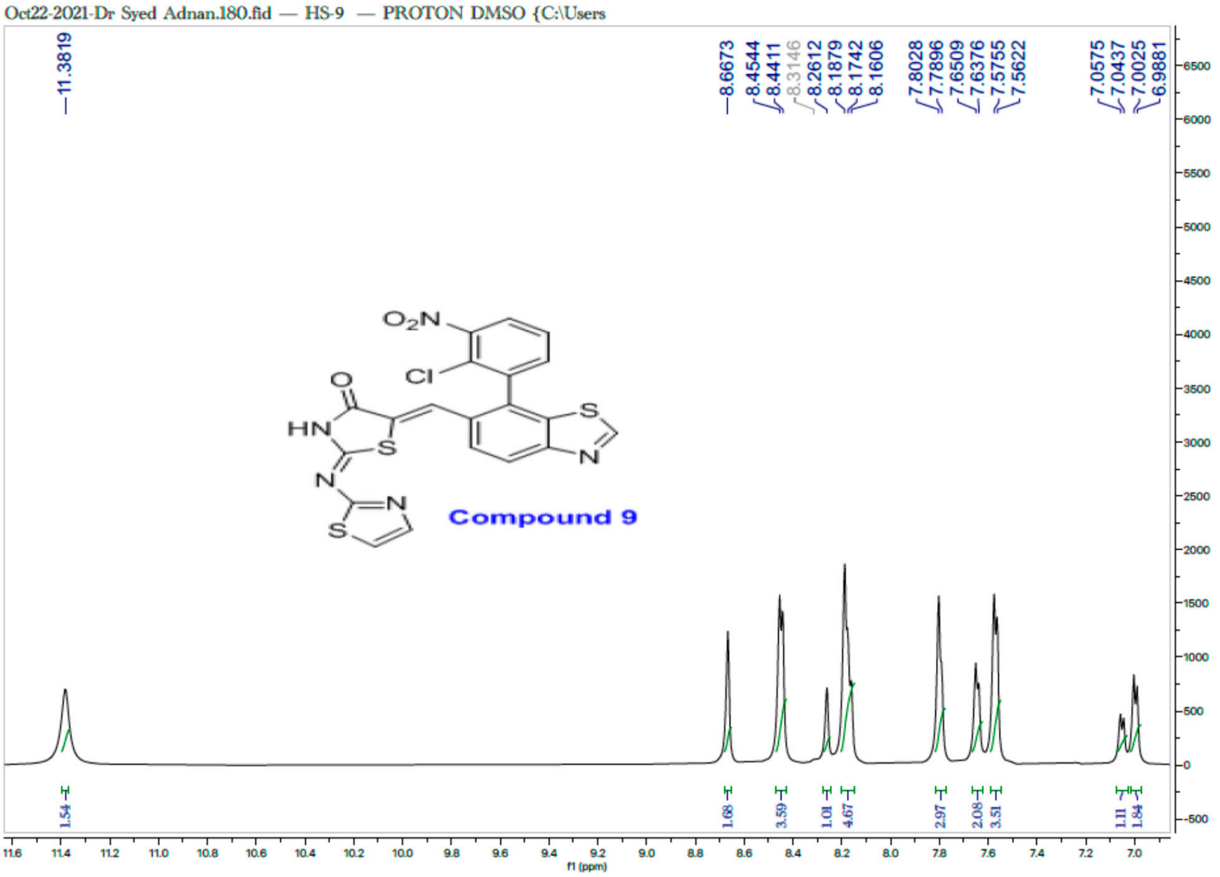

Figure S3. High resolution Proton NMR Spectra of (2Z,5Z)-5-((7-(2-chloro-3-nitrophenyl)benzo[d]thiazol-6-yl)methylene)-2-(thiazol-2-ylimino)thiazolidin-4-one (9)

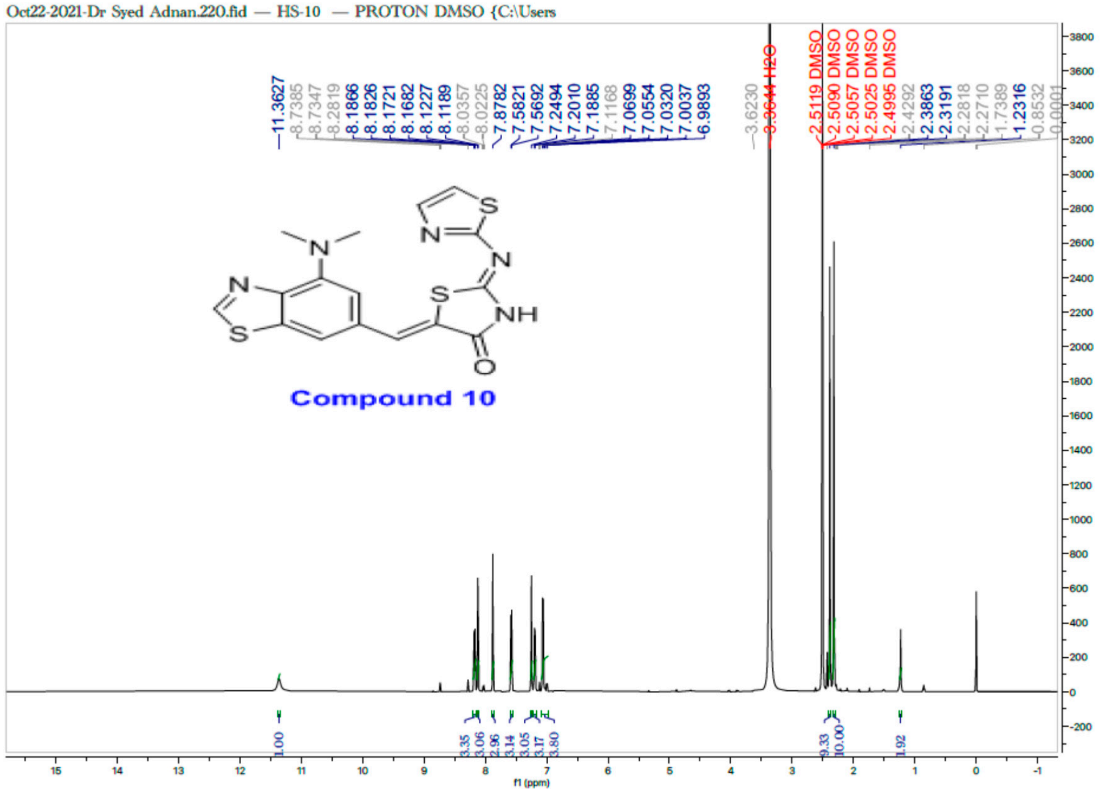
Oct22-2021-Dr Syed Adnan.220.fid — HS-10 — PROTON DMSO {C:\Users
Compound 10
f1 (ppm)

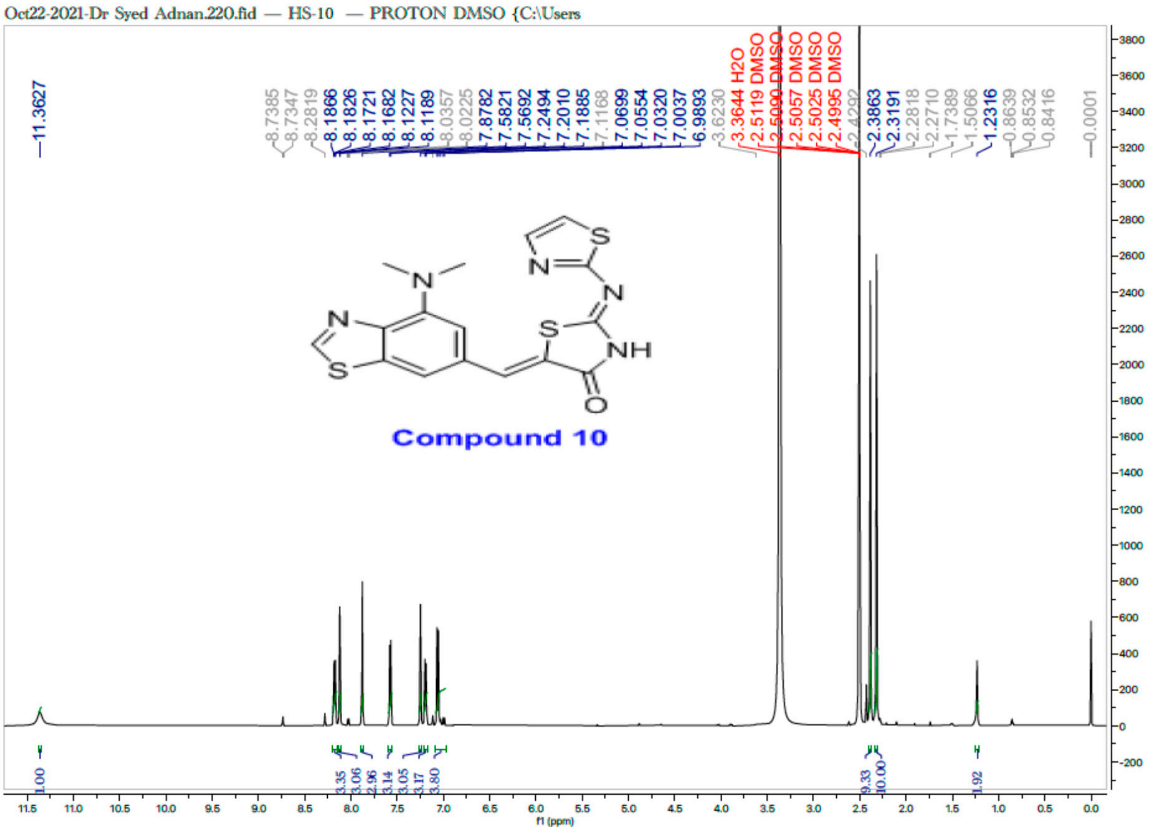
Oct22-2021-Dr Syed Adnan.220.fid — HS-10 — PROTON DMSO {C:\Users
Compound 10
f1 (ppm)

Figure S4. **Low resolution Proton NMR Spectra of (2Z,5Z)-5-((4-(dimethylamino)benzo[d]thiazol-6-yl)methylene)-2-(thiazol-2-ylimino)thiazolidin-4-one (10)**

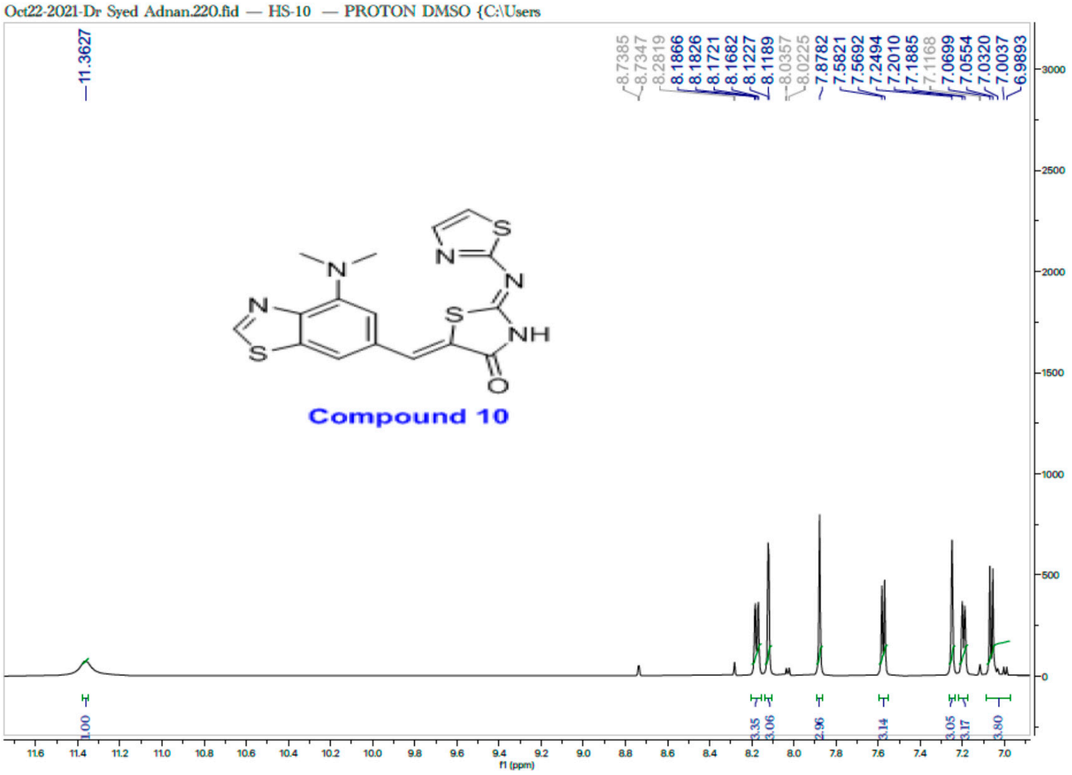
Oct22-2021-Dr Syed Adnan.220.fid — HS-10 — PROTON DMSO {C:\Users
11.3627
8.7385
8.7347
8.2819
8.1866
8.1826
8.1721
8.1682
8.1227
8.1189
8.0357
8.0225
7.8782
7.5821
7.5692
7.2494
7.2010
7.1885
7.1168
7.0699
7.0554
7.0320
7.0037
6.9893
Compound 10
1.00
3.35
3.06
2.96
3.14
3.05
3.17
3.80
f1 (ppm)

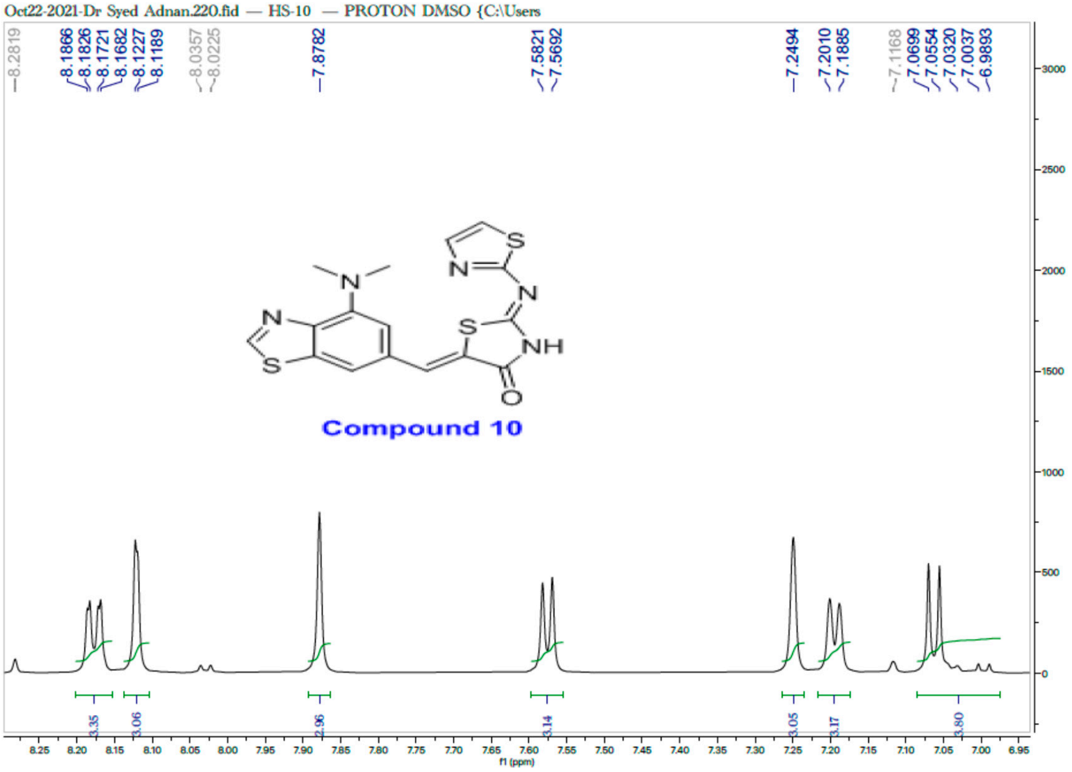
Oct22-2021-Dr Syed Adnan.220.fid — HS-10 — PROTON DMSO {C:\Users
8.2819
8.1866
8.1826
8.1721
8.1682
8.1227
8.1189
8.0357
8.0225
7.8782
7.5821
7.5692
7.2494
7.2010
7.1885
7.1168
7.0699
7.0554
7.0320
7.0037
6.9893
Compound 10
3.35
3.06
2.96
3.14
3.05
3.17
3.80
f1 (ppm)

Figure S5. **High resolution Proton NMR Spectra of (2Z,5Z)-5-((4-(dimethylamino)benzo[d]thiazol-6-yl)methylene)-2-(thiazol-2-ylimino)thiazolidin-4-one (10)**

Oct22-2021-Dr Syed Adnan.80.fid — HS-15 — PROTON DMSO {C:\Users

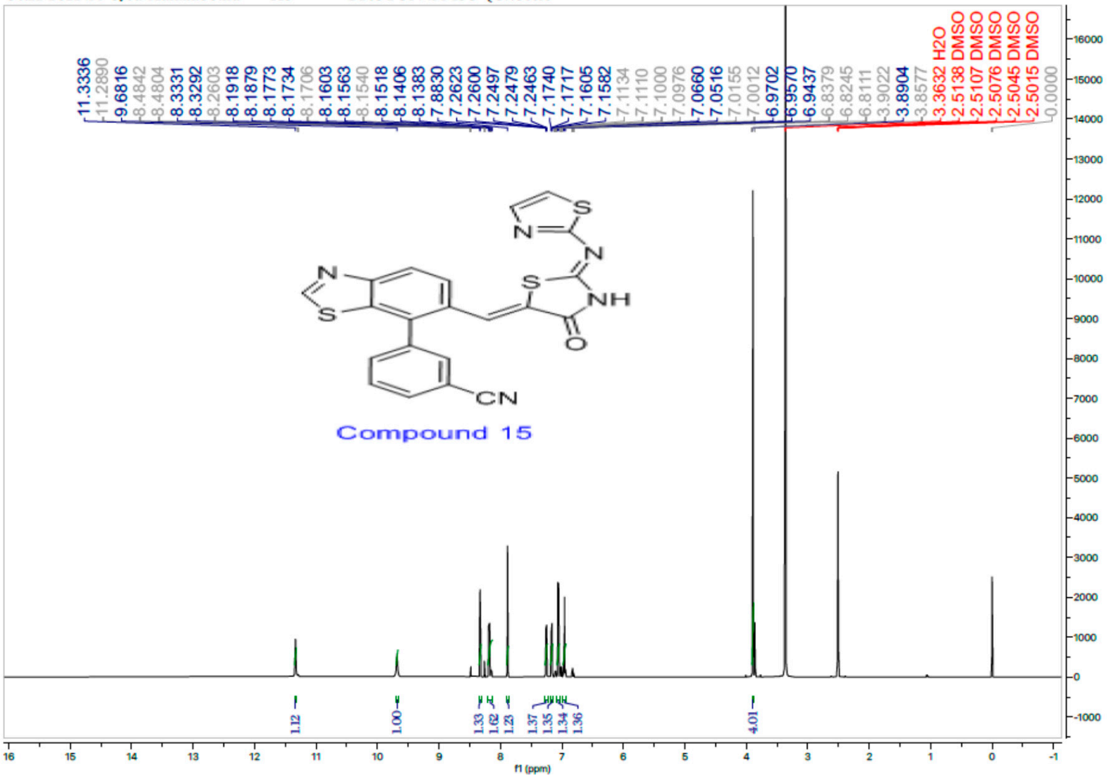

Oct22-2021-Dr Syed Adnan.80.fid — HS-15 — PROTON DMSO {C:\Users

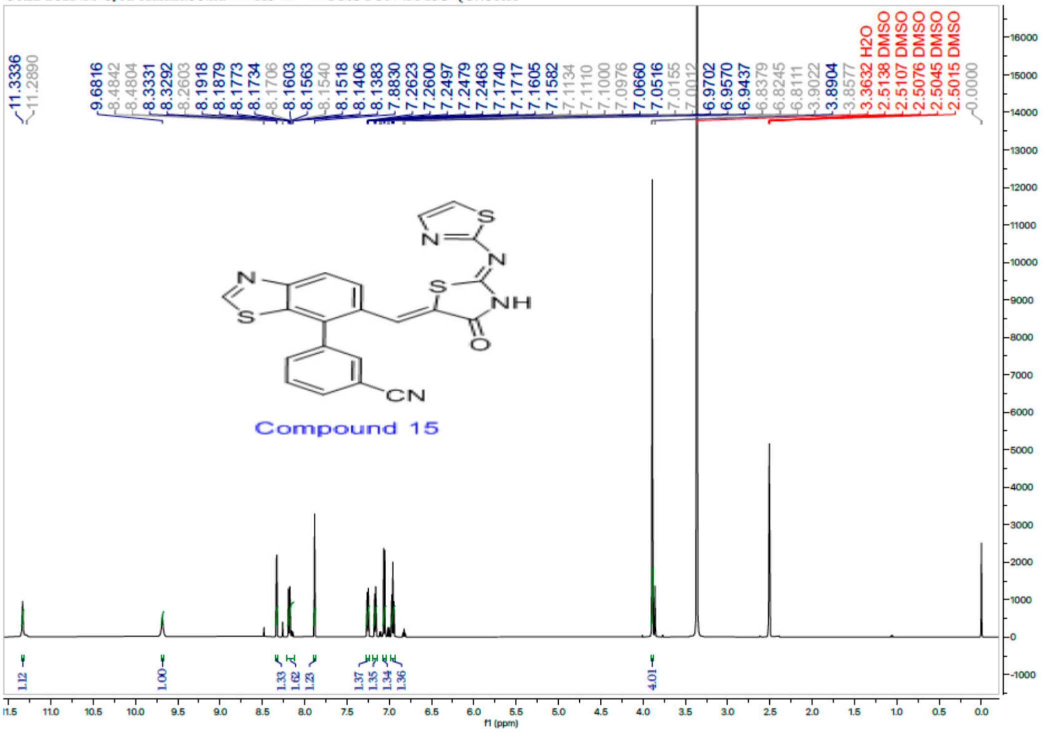

Figure S6. Low resolution Proton NMR Spectra of 3-(6-((Z)-((Z)-4-oxo-2-(thiazol-2-ylimino) thiazolidin-5-ylidene)methyl)benzo[d]thiazol-7-yl)benzonitrile (15)

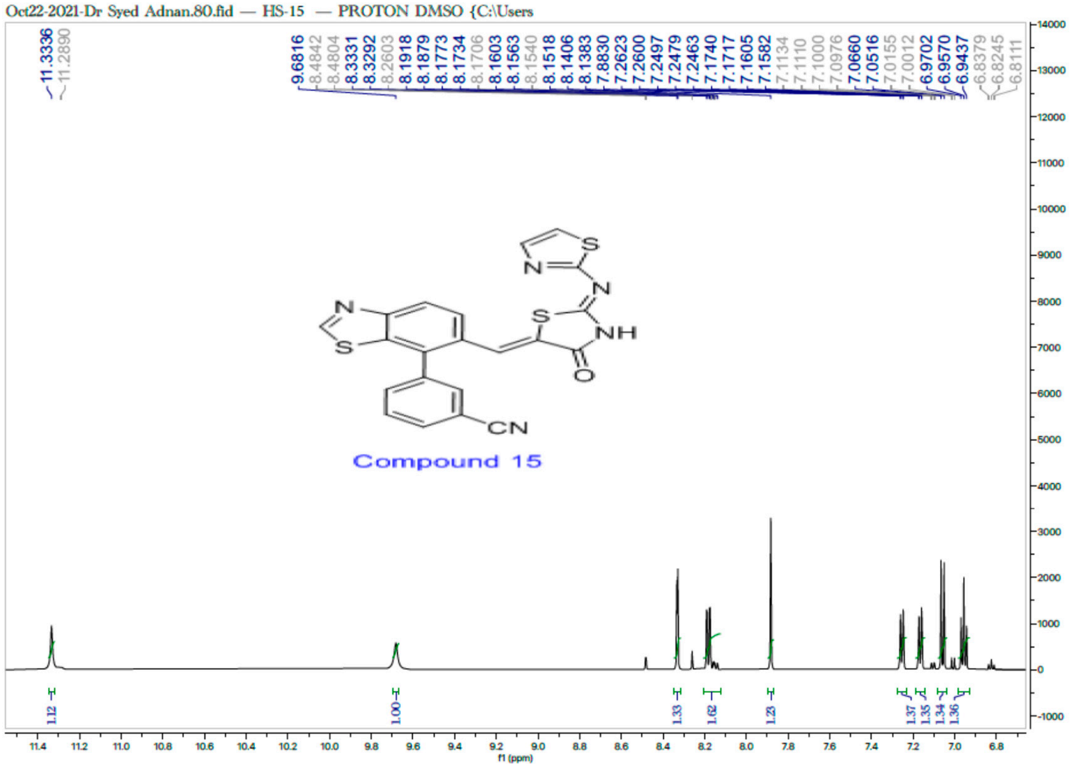
Oct22-2021-Dr Syed Adnan.80.fid — HS-15 — PROTON DMSO {C:\Users
Compound 15
CN
NH
f1 (ppm)

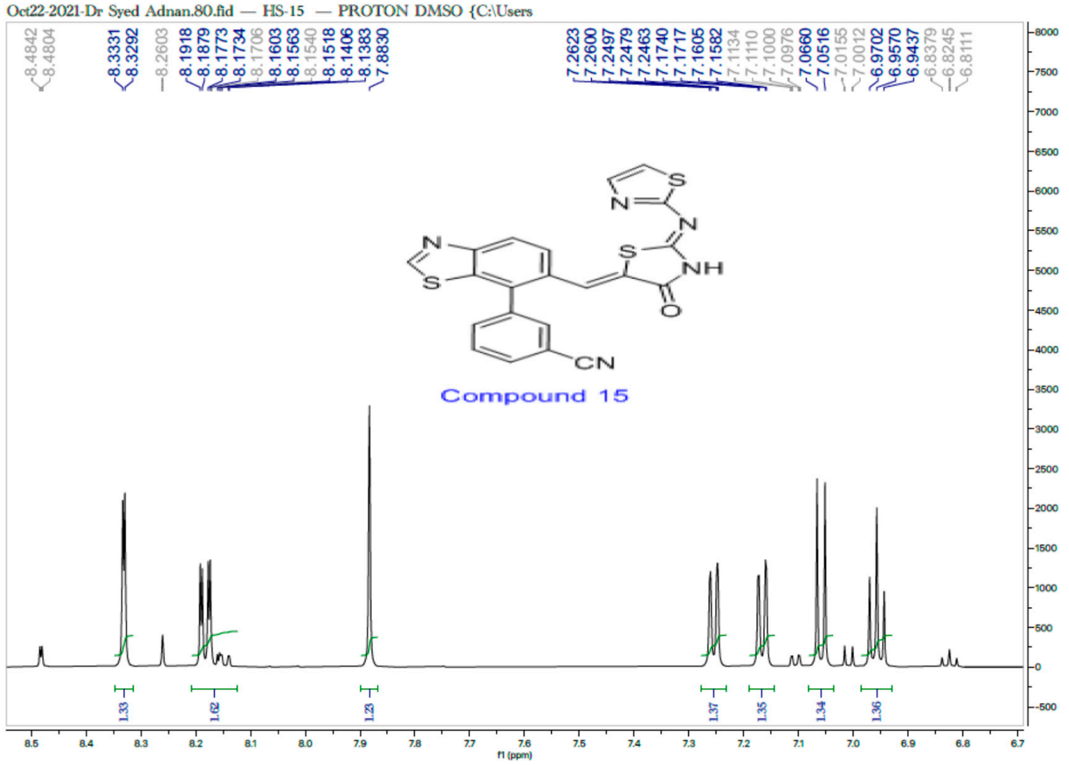
Oct22-2021-Dr Syed Adnan.80.fid — HS-15 — PROTON DMSO {C:\Users
Compound 15
CN
NH
f1 (ppm)

Figure S7. **High resolution Proton NMR Spectra of 3-(6-((Z)-((Z)-4-oxo-2-(thiazol-2-ylimino) thiazolidin-5-ylidene)methyl)benzo[d]thiazol-7-yl)benzonitrile (15)**

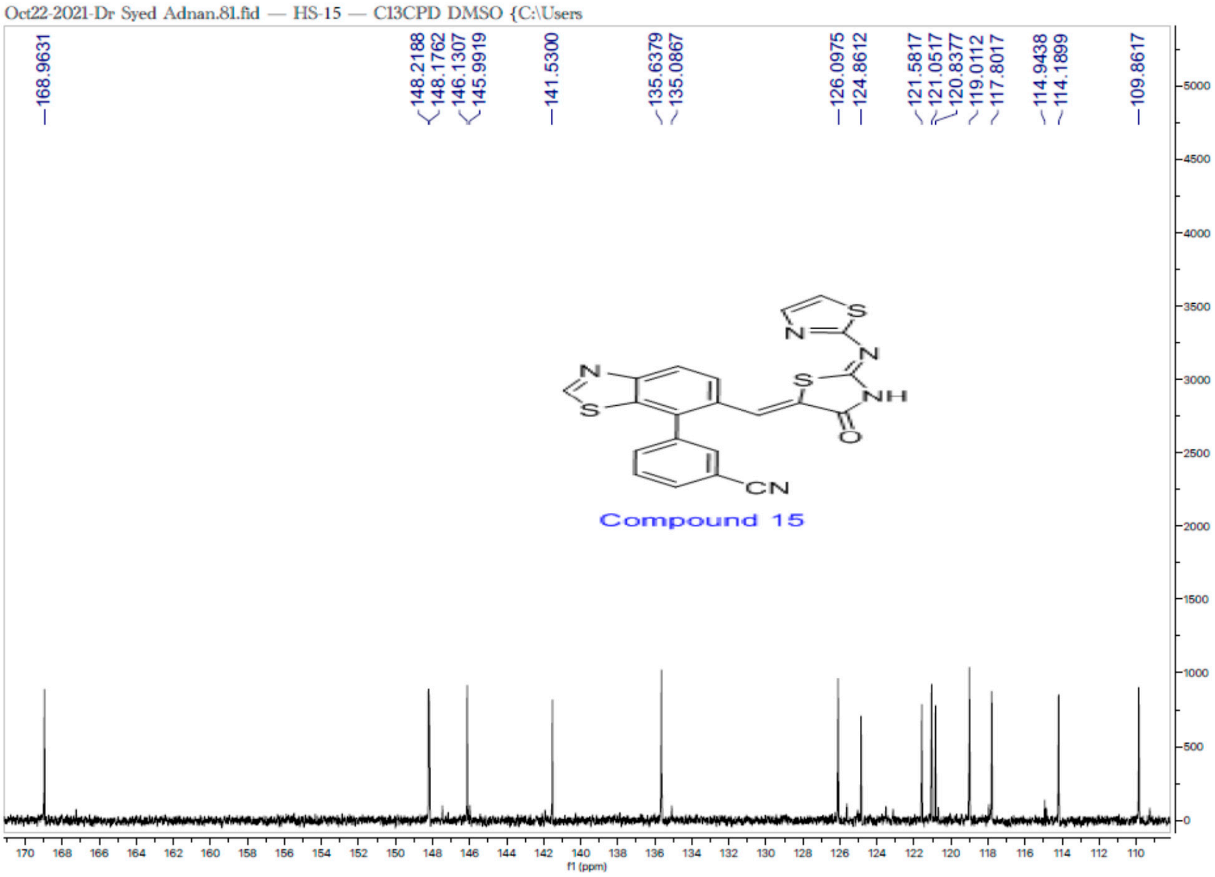

Figure S8. **High resolution 13C-NMR Spectra of 3-(6-((Z)-((Z)-4-oxo-2-(thiazol-2-ylimino)thiazolidin-5-ylidene)methyl)benzo[d]thiazol-7-yl)benzonitrile (15)**

References

[1] Srinivasa, M.G.; Aggarwal, N.N.; Gatpoh, B.F.D.; Shankar, M.K.; Byadarahalli Ravindranath, K.; Gurubasavaraj Veeranna, P.; Dixit, S.; Mandal, S.P.; Bommenahally Ravanappa, P.K.; Khanal, P.; et al. Identification of benzothiazole−rhodanine derivatives as α-amylase and α-glucosidase inhibitors: Design, synthesis, in silico, and in vitro analysis. *J. Mol. Recognit.* **2022**, *35*, e2959.

[2] Khan, S.; Ullah, H.; Rahim, F.; Nawaz, M.; Hussain, R.; Rasheed, L. Synthesis, in vitro α-amylase, α-glucosidase activities and molecular docking study of new benzimidazole bearing thiazolidinone derivatives. *J. Mol. Struct.* **2022**, *1269*, 133812.
